# A Digital Atlas of Ion Channel Expression Patterns in the Two-Week-Old Rat Brain

**DOI:** 10.1007/s12021-014-9247-0

**Published:** 2014-10-07

**Authors:** Volodymyr Shcherbatyy, James Carson, Murat Yaylaoglu, Katharina Jäckle, Frauke Grabbe, Maren Brockmeyer, Halenur Yavuz, Gregor Eichele

**Affiliations:** 1 Department of Genes and Behavior, Max Planck Institute for Biophysical Chemistry, Am Fassberg 11, 37077 Göttingen, Germany; 2 Life Sciences Computing, Texas Advanced Computing Center, 10100 Burnet Road, Austin, TX 78758 USA

**Keywords:** Ion channels, Gene expression analysis, In situ hybridization, Genepaint.org database, Rat brain, Digital atlas, Subdivision mesh

## Abstract

The approximately 350 ion channels encoded by the mammalian genome are a main pillar of the nervous system. We have determined the expression pattern of 320 channels in the two-week-old (P14) rat brain by means of non-radioactive robotic in situ hybridization. Optimized methods were developed and implemented to generate stringently coronal brain sections. The use of standardized methods permits a direct comparison of expression patterns across the entire ion channel expression pattern data set and facilitates recognizing ion channel co-expression. All expression data are made publically available at the Genepaint.org database. Inwardly rectifying potassium channels (Kir, encoded by the *Kcnj* genes) regulate a broad spectrum of physiological processes. *Kcnj* channel expression patterns generated in the present study were fitted with a deformable subdivision mesh atlas produced for the P14 rat brain. This co-registration, when combined with numerical quantification of expression strengths, allowed for semi-quantitative automated annotation of expression patterns as well as comparisons among and between *Kcnj* subfamilies. The expression patterns of *Kcnj* channel were also cross validated against previously published expression patterns of *Kcnj* channel genes.

**Electronic supplementary material:**

The online version of this article (doi:10.1007/s12021-014-9247-0) contains supplementary material, which is available to authorized users.

## Introduction

Ion channels arguably are the functionally most important proteins of the nervous system. Accordingly, there exists a wealth of studies illustrating their spatiotemporal expression patterns at mRNA and protein levels. Critical knowledge about expression patterns includes information on co-expression of channel auxiliary subunits that form oligomers and co-expression of channels known to operate in a concerted fashion. These requirements necessitate that expression data be placed into a common reference frame, either computationally by registering data from different published studies or by redetermination of expression patterns of all ion channels (channelome) in a standardized manner. The latter approach not only affords a systematic quantification of expression strength but, when data are appropriately collected, the resulting information can be placed into a digital, searchable atlas, which allows one to compare and contrast expression patterns. The Allen Brain Atlas of the adult mouse brain provides a first example of how this goal can be realized on a large scale (Lein et al. 2007).

Juvenile rodent brains are frequently used for physiological studies in slice cultures (Fuller and Dailey 2007). Therefore, we determined the expression patterns of the channelome in the two-week-old rat brain and placed this information into Genepaint.org, a searchable web-based atlas (http://www.genepaint.org/). To compare expression patterns from different specimens, we implemented the following measures. First, we developed a highly reproducible method of brain orientation and sectioning. Second, we designed templates of similar length for 320 ion channels to synthesize non-radioactive riboprobes that we then used to determine the expression patterns by robotic in situ hybridization (ISH). Third, we registered a selected subset of these expression data with a multi-resolution deformable atlas to annotate expression patterns and assess the similarity of sections from different brains. Subdivision meshes enable explicit modeling of anatomical region boundaries and smoothly represent a coordinate space within each region (Ju et al. 2010). Lastly, we selected the inwardly rectifying potassium channels for a comparison of our expression results with those seen in previously published manuscripts and digital resources.

Inwardly rectifying K^+^ channels (Kir) are required for a diverse array of physiological processes including the maintenance of K^+^ homeostasis, transepithelial ion flux, hormone secretion, heart rate modulation, neurogenesis and differentiation in the central nervous system (Nichols and Lopatin 1997; Reimann and Ashcroft 1999; Miki et al. 2001; Lu 2004). Ectopic expression, crystallographic and electrophysiological studies have led to detailed structural and kinetic models of Kir channels that reveal the molecular basis of channel activity (Ho et al. 1993; Kubo et al. 1993; Nishida and MacKinnon 2002; Yu and Catterall 2004). Kir channel subunits form a tetrameric complex and each of the subunits contains two transmembrane segments (TM1 and TM2) along with a pore-forming (H5) loop. There are 15 members of the Kir family, which is composed of seven subfamilies (Kir1 to Kir7) (Kubo et al. 2005) that can be classified into four functional groups (Hibino et al. 2010). Kir channels have the ability to form functional channels from both homomeric and heteromeric assemblies. Heteromeric channels are generally formed by co-assembly of subunits with other members of the same subfamily (Krapivinsky et al. 1995; Schram et al. 2002; Preisig-Muller et al. 2002). However, heteromeric assembly beyond the subfamily have been demonstrated (Pessia et al. 2001; Hibino et al. 2004; Ishihara et al. 2009).

Kir channels in humans are encoded by the *KCNJ* genes, and these in turn are associated with channelopathies (Abraham et al. 1999; Neusch et al. 2003; Benarroch 2009). Mutations of Kir channels can cause Bartter’s syndrome type II (*KCNJ1*) (Bartter et al. 1962; Naesens et al. 2004), Andersen’s syndrome (*KCNJ2*) (Andersen et al. 1971; Bendahhou et al. 2003; Donaldson et al. 2003), and persistent hyperinsulinemic hypoglykemia of infancy and/or neonatal diabetes (*KCNJ11*) (Ashcroft 2005; Shimomura 2009). Moreover, Kir subunits are promising candidates for trisomy 21 phenotypes (e.g. *KCNJ15*) (Gosset et al. 1997; Reymond et al. 2002; Thiery et al. 2003), and the *weaver* phenotype in mice has been linked to a *Kcnj6* mutation (Patil et al. 1995). The importance of understanding Kir channels is further highlighted by their role as potential drug targets for therapeutic treatments of various diseases and pathological conditions (Judge et al. 2007; Sun and Hu 2010).

Taken together, the present study creates a comprehensive and standardized digital compendium of ion channels expression patterns at cellular resolution. This information is helpful for the planning of physiological studies and for the genetic manipulation of individual ion channels.

## Materials and Methods

### Tissue Collection and Sectioning

Postnatal day 14 (P14) Wistar rats (weight ~24.5 g) were sacrificed by decapitation under Isoflurane (Baxter) anesthesia. Each dissected brain was then individually transferred to a custom-made freezing chamber (Eichele and Diez-Roux 2011) filled with optimum cutting temperature medium (OCT, Sakura Finetek) and frozen as previously described for mouse brains (Yaylaoglu et al. 2005). However, the freezing chambers used for rat brains were larger, having internal dimensions of 22 × 22 × 22 mm. The OCT-embedded fresh frozen brains were sectioned coronally on a Leica cryostat Model CM 3050S with a section thickness of 25 μm. Each brain was cut into 8 serial sets of 10 slides with each slide containing 3 sections. This leads to a spacing of 200 μm between sections within a set and 30 sections per set. Set number 4 was Nissl-stained for use as an anatomical reference and for quality control of sectioning. The remaining seven sets were used for ISH. Sections were fixed in 4 % paraformaldehyde, acetylated in 0.25 % acetic anhydride and dried in an ethanol series in a Leica Autostainer XL as described previously (Yaylaoglu et al. 2005).

### RNA Probe Synthesis, RNA ISH and qPCR

Riboprobe preparation and automated ISH were performed as previously described (Yaylaoglu et al. 2005; Eichele and Diez-Roux 2011). RNA was extracted from whole rat brain and used to synthesize cDNA by reverse transcription. The cDNA was then used for synthesizing specific templates for each ion channel gene by selecting a unique region for each gene (template sequences for each gene are accessible at Genepaint.org; see Online Resource 1 for more details). Templates were in vitro transcribed in the presence of digoxygenin-tagged UTPs to generate digoxygenin-tagged RNA probes. Robotic non-radioactive ISH was performed on slides containing collected cryosections using these probes.

Serial qPCR experiments were executed to validate relative expression levels of 128 from the 320 ion channel genes in brain, kidney, thymus and lung of P14 rat. RNA samples were isolated from three different animals. The quality of these extracts was checked on the Bioanalyzer (Agilent) and cDNAs were synthesized. PCR reactions were carried in an iCycler (BioRad) as previously described (Yaylaoglu et al. 2005). For primer sequences, see Online Resource 1.

### Image Acquisition and Annotation of Expression Pattern

After robotic ISH, slides were cover-slipped and digitally imaged at 1.6 μm/pixel using an automated Leica DM-RXA2 microscope (Carson et al. 2002). These images were automatically cropped in Adobe Photoshop and stored as TIFF files with LZW lossless compression. TIFFs were uploaded onto the Genepaint.org database where they can be viewed interactively. For *Kcnj* genes, expression strength and pattern was assigned in 13 major brain regions (cerebral cortex, hippocampus, caudate putamen, globus pallidus, basal forebrain, septum, amygdala, thalamus, hypothalamus, midbrain, pons, ventricles and fiber tracts) using an established atlas-based registration approach (Carson et al. 2004). This annotation approach leverages *celldetekt* software which classifies the spatial area of dye precipitate in each cell resulting from colorimetric detection (Carson et al. 2005a) to annotate using traditional semi-quantitative descriptions of pattern (regional, scattered, ubiquitous) and strength (strong, moderate, weak, none). Pattern assignment is based on the total percentage of cells expressing the mRNA within a region and the scaled weighted deviation in the percentage of cells expressing the gene across the atlas subunits within the structure (Carson et al. 2004).

## Results

First we established a method allowing the production of well-oriented sections from the postnatal rat brain suitable for robotic ISH. Following validation of this procedure we applied it to determine the expression patterns of the rat channelome in P14 rat brain. Next, we generated a subdivision mesh atlas of the P14 rat brain that was registered to the experimental sections from the *Kcnj* channel gene family in order to systematically annotate, compare and interrogate expression patterns, even between different brain specimens. The quality of the experimental data, the reliability of matching experimental data with the subdivision mesh atlas, and consistency with published work were assessed for *Kcnj* channel gene family.

### Production of Oriented Coronal Sections

**Fig. 1:**
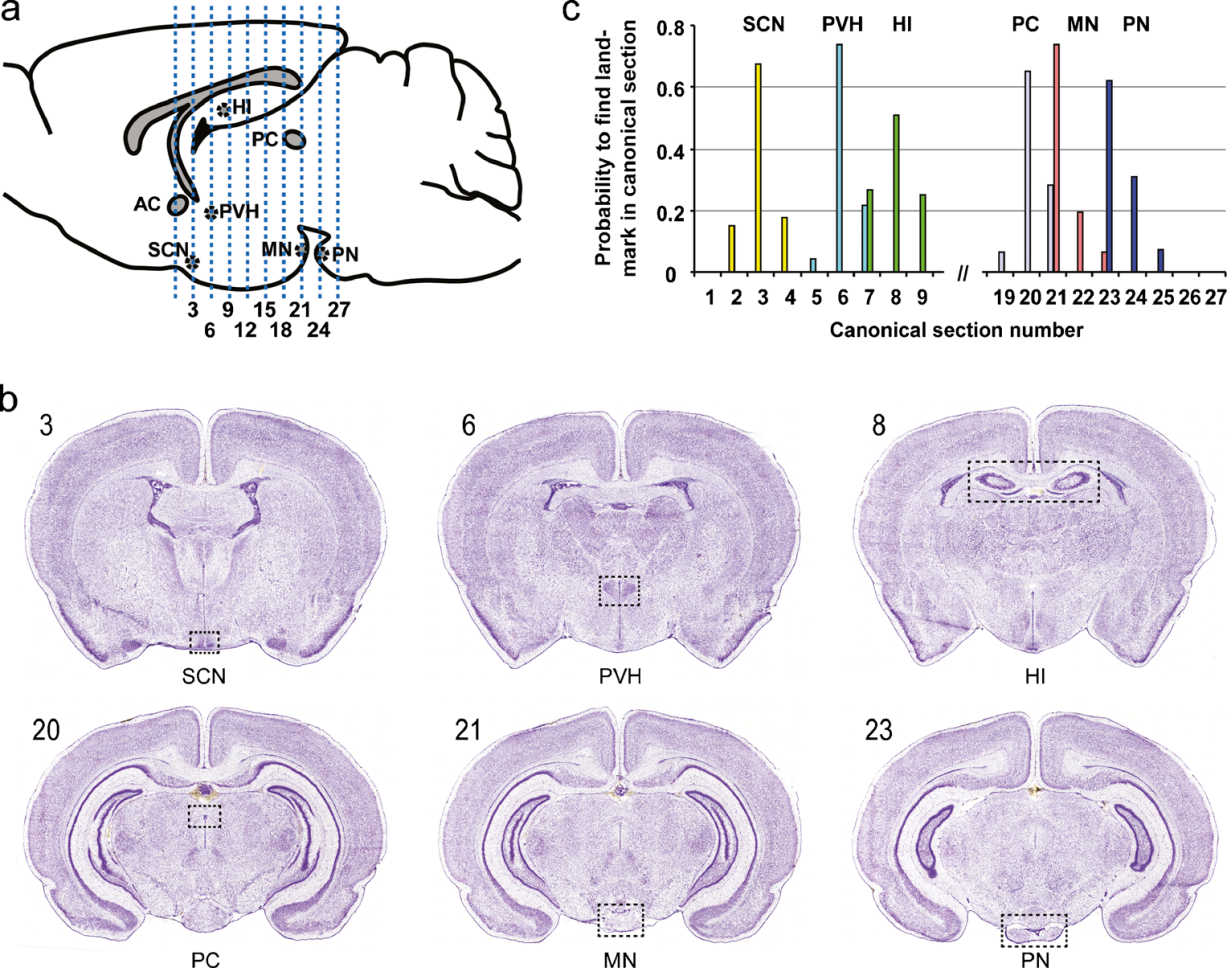
Location of anatomical landmarks. **a** Positions of the 27 section planes along the rostrocaudal axis. **b** Images of Nissl-stained canonical sections with landmarks indicated. **c** Distribution of landmarks in 43 different brains. *Abbreviations*: *AC* anterior commissure, *HI* hippocampus, *MN* mammillary nucleus, *PC* posterior commissure, *PN* pontine nucleus, *PVH* paraventricular nucleus of the hypothalamus, *SCN* suprachiasmatic nucleus

To ensure that expression patterns from different brains can be directly compared, we sectioned using the following approach. Brains were placed ventral side up in OCT-filled, custom-made freezing chambers (see Methods). Sectioning began at the rostral end and periodically sample sections were collected and stained with methylene blue until the level corresponding to the line drawing of Fig. 26 (Bregma 0.84 mm) in “The Rat Brain in Stereotaxic Coordinates” (Paxinos and Watson 2013) was reached. The stained anatomical structures guided specimen adjustment in the coronal plane with the assistance of the goniometer head of the cryostat. Collection of sections sets was initiated in the plane containing the merger of the two branches of the anterior commissure (Fig. 33 [Bregma 0.00 mm] in Paxinos and Watson 2013). Sectioning was terminated after collecting 240 sections (approximately Fig. 90 [Bregma 6.84 mm] in Paxinos and Watson 2013). Section set 4 was Nissl-stained and assessed for the presence of six anatomical landmarks: the suprachiasmatic nucleus (SCN, section plane #3), the paraventricular nucleus of the hypothalamus (PVH, section plane #6), the anterior edge of the hippocampus (section plane #8), the posterior commissure (section plane #20), the mammillary nucleus (section plane #21), and the pontine nucleus (section plane #23) (Fig. 1a, b). Only the brains for which these six landmarks were either in the canonical section plane (as defined above), or in the preceding or subsequent section, were further processed for ISH. Figure 1c provides a graphical illustration of the distribution of landmarks across coronal planes for brains utilized for ISH. For the majority of specimens, the six landmarks were found in the canonical section plane, while for the remainder, the landmarks were found either in the preceding or following section. Because the distance between the sections of a set was 0.2 mm and the total length of the rat P14 brain is approximately 15 mm, the divergence between sets is in the range of 1 to 2%. We conclude that our approach is sufficiently accurate to allow for a direct comparison of expression patterns of different genes from different brains.

### Rat Channelome

There are approximately 350 ion channels in mammalian genomes and we were able to generate templates for 320 of them for subsequent ISH. Table 1 provides an overview of the rat channelome families and summarizes our ISH and qPCR data. Online Resource 1 provides more detailed information for each individual channel gene. Table 1 shows for each channel gene family the number of members that are either detected or not detected by ISH. It can readily be seen that most ion channels, irrespective of the family, are expressed.

**Table 1:** Overview of data collected for the rat channelome in P14 rat brain

Ion channel family	Number of members	ISH Data		qPCR Analysis	
		Number of members analyzed and Detected	Number of members analyzed but not Detected	Number of members analyzed and Detected	Number of members analyzed but not Detected
Voltage-gated ion channels					
Calcium channels	26	24	1	1	
Cyclic nucleotide gated channels	10	6	2	4	
Potassium channels	49	49	1		
Inwardly-Rectifying Potassium Channels	15	15	1		
Two-P Potassium Channels	14	13	1		
Calcium-Activated Potassium Channels	12	9	2	2	
Sodium channels	14	14	1		
CatSper and Two-Pore channels	6	3	3	3	
Transient receptor potential channels	29	20	4	24	5
Anion channels	3	3	3		
Subtotal	178	156	5	37	16
Ligand-Gated Ion Channels					
Amiloride receptors	8	5	6	2	
Nicotinic acetylcholine receptors	16	15	1		
Dopamine receptors	5	5	1		
GABA-A receptors	20	19	1	1	
Glycine receptors	5	5			
Glutamate receptors	27	27	1		
5-HT3 (Serotonin) receptors	13	12	1		
Inositol receptors	3	3	3		
Purinergic receptors P2X	7	7	5		
Ryanodine receptors	3	3			
Other Ligand-Gated Channels	3	2	2	1	
Subtotal	110	103	19	6	
Other ion channels					
Chloride channels	20	14	8	3	
Other potassium channels	25	19	22	2	
Auxiliary subunits	24	23	14	1	
Subtotal	69	56	44	6	
TOTAL ion channels	357	315	5	100	28

Some of the ISH data were cross-validated with qPCR analysis (Table 1 and Online Resource 1) because in the course of testing channel riboprobes on brain sections, several yielded either weak signal or appeared not to be detectable. These channels were then subjected to qPCR with brain-derived cDNA and as controls also with cDNA from thymus, lung and kidney. We found that the 28 channels for which the qPCR analysis was negative in brain (Table 1 and Online Resource 1) were mostly those for which we were not able to make a template for ISH from brain cDNA. Also note that of the 28 negative cases in brain, 17 were positive in one or more of the other tissues examined. Most of the 11 channels that had escaped detection by either ISH or qPCR are associated with specific cell types such as sperm, smooth muscle and retinal photoreceptors, so that their expression was not to be expected in brain.

The ISH expression patterns of 320 channel genes were deposited into Genepaint.org. Genepaint.org database also contains expression patterns in the mouse embryo (Visel et al. 2004) of many of the mouse orthologs of rat channel genes. This provides access to information about the developmental expression patterns of ion channels. Since the embryo contains many non-neuronal tissues, channel expression in such tissues can also be inspected.

### Construction of a Subdivision Mesh Atlas of the P14 rat Brain

**Fig. 2:**
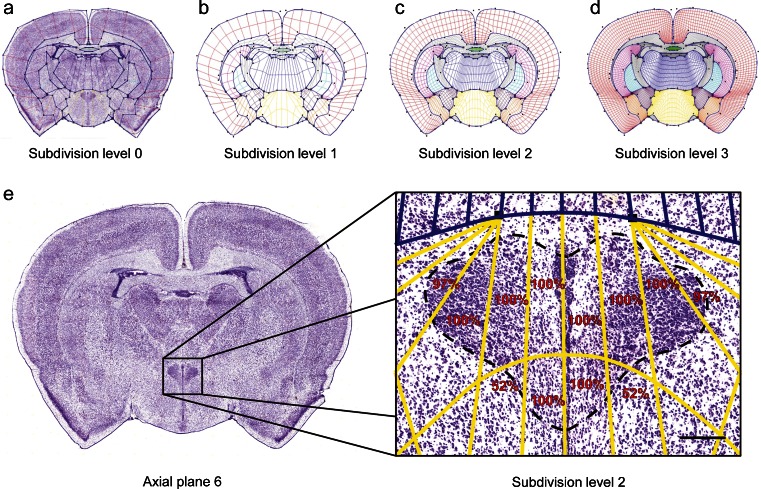
Principles of subdivision mesh atlas construction. **a** Nissl-stained section at axial plane 6 superimposed with the subdivision mesh at level 0. **b**–**d** Depict how the mesh further subdivides at levels 1, 2 and 3, respectively. **e** Accuracy of mesh atlas. Twelve quadrilaterals encompass the entire PVH at mesh atlas plane 6. The same eight quadrilaterals overlap the expanse of the PVH (100 %) for all 31 specimens examined, while the quadrilaterals at the edge score 52 or 97 %, respectively. Scale bar = 200 μm

While the Genepaint.org database provides easy access to the expression patterns of channel genes in the postnatal rat brain, sites and levels of expression are not annotated and therefore a systematic comparison between sections and genes cannot be done. To make this possible, we constructed a subdivision mesh atlas for the P14 rat brain following the strategy described previously (Carson et al. 2005b). Subdivision meshes are inherently multiresolution, with each application of subdivision dividing every quadrilateral of the mesh into four smaller quadrilaterals, a process that increments the “subdivision level” by 1 (Warren and Weimer 2002). Of note, currently the atlas is restricted to the portion of the brain for which sections were collected and therefore includes most of the fore- and midbrain. A total of 27 standard atlas meshes were constructed with each standard mesh corresponding to one of the experimental sections for which ISH data were collected. Figure 2a shows a Nissl-stained section of plane 6 and the associated standard mesh. Meshes are fitted to the ISH sections by a simple manual drag-and-drop of “control points” marked by a black square, thus allowing for precise and accurate placement of the boundaries depicting the 13 major regions of the brain. On rare instance, generally due to a small tear during sectioning, a very small fraction of tissue will extend beyond the atlas boundaries. Online Resource 2 depicts all 27 meshes of the P14 rat brain atlas.

We next assessed the variation of the position of the PVH in the subdivision mesh atlas. For this we selected a total of 31 experimental sections at axial plane 6 which contain the PVH most frequently (Fig. 1b and c) and then determined which quadrilaterals of the mesh atlas at subdivided level 2 coincided with this nucleus. We found that the PVH was fully contained within the 12 quadrilaterals for the majority of specimens. In every one of the 31 sections, the same 8 quadrilaterals overlap with the PVH (100 % in Fig. 2e), whereas 4 adjacent quadrilaterals covered only the PVH’s edges. Therefore, the atlas is sufficiently accurate to afford a direct comparison of sections from brains of different animals.

Taken together, we established a subdivision mesh atlas covering many of the key structures of the rat brain. This atlas when fitted to ISH sections can now be used for annotating and interrogating gene expression patterns.

### Detailed Analysis of *Kcnj* Channel Genes

We compared our expression data with previously published results and applied the subdivision mesh atlas for systematic gene expression analysis. We focused on *Kcnj* channel genes that form a small, but important family of ion channels with diverse physiological roles (see Introduction). This expression analysis consisted of two steps. First we applied, section by section, the quantification software *celldetekt* to the ISH data and subsequently fit subdivision meshes to each of the sections. By combining these two steps, we generated quantitative expression strength information for each quadrilateral in the subdivided standard mesh. Based on these data, we computed a report of expression strength and pattern for each of the main brain structures (Table 2) as previously described [see Fig. 6 in Carson et al. (2010)]. We found that expression patterns of *Kcnj* genes were quite diverse. Some *Kcnj* were concentrated to specific brain regions (regional, “R”, e.g. *Kcnj13* in the ventricles and *Kcnj5* in cortex), while others were expressed broadly in a particular structure (ubiquitous, “U”, e.g. *Kcnj6* in caudate putamen). The frequently occurring “S” in Table 2 stands for “scattered” which are patterns where a single cell or a cluster of cells expresses a particular gene while surrounding cells do not, e.g. signal is restricted to a subpopulation of cells (i.e. neurons, interneurons, astro- and/or oligodendrocytes or vascular cell-type). Levels of expression are also variable and are represented by the attributes “-“, “+”, “++” and “+++” (none, weak, moderate, strong) as well as the colors grey, yellow, blue and red, respectively.

**Table 2:**
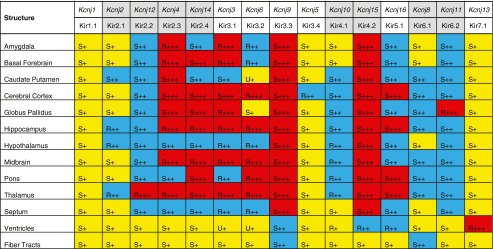
Patterns annotated by gene and structure for *Kcnj* channel family

*R* regional, *S* scattered, *U* ubiquitous; +++ strong (*red*); ++: moderate (*blue*); +: weak (*yellow*)

**Fig. 3:**
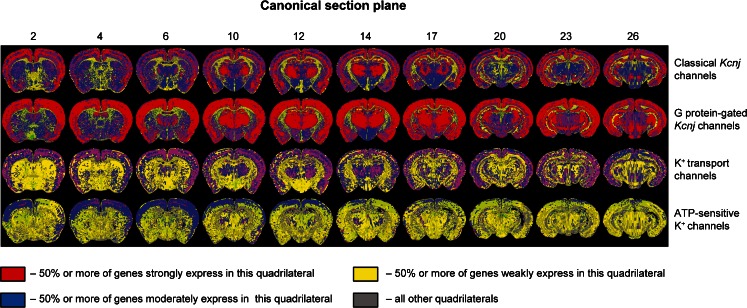
Heatmap of the expression patterns of the *Kcnj* channel genes. Functional groups: Classical *Kcnj* channels (*Kcnj2*, *4*, *12 and 14*); G protein-gated *Kcnj* channels (*Kcnj3*, *5*, *6 and 9*); K^+^ transport channels (*Kcnj1*, *10*, *13*, *15 and 16*) and ATP-sensitive K^+^ channels (*Kcnj8*, *11 and Abcc8*, *9*). Note that the color maps emphasize that the four functional groups have a markedly different expression pattern

Kir channel subunits from within a subfamily or functional group (Classical *Kcnj* channels, G protein-gated *Kcnj* channels, ATP-sensitive K^+^ channels and K^+^ transport channels) can heteromerize to form functional Kir channels (see Introduction). Quantification of expression within the subdivision mesh allows for the graphical representation of sites of co-expression of several channel genes (Fig. 3). For example, *Kcnj3*, *5*, *6* and *9* encode the G protein-gated channel subfamily (Fig. 3, second row). As can be seen, the corresponding mRNAs are strongly expressed in the cortex, hippocampus (section planes #10-26), thalamus (section planes #6-26) and the substantia nigra (section planes #23-26). In contrast, transcript encoding ATP-sensitive potassium channels (*Kcnj8* and *11*) and their auxiliary subunits (*Abcc8* and *9*) are characterized by an overall weaker expression (Fig. 3, bottom row).

While global reports (Table 2 and Fig. 3) provide a useful overview of *Kcnj* gene expression motifs, the very detailed information innate to the ISH data reveal many more features of the expression patterns for each individual *Kcnj* channel gene. In what follows we discuss the main aspects of the expression patterns of *Kcnj* channels, but the reader is encouraged to consult the Genepaint.org database where the original data can be inspected at full resolution and in color. To facilitate access to these data we provide the Genepaint set ID in the text below.

### Classical *Kcnj* Channels (Kir2.x)

#### *Kcnj2*/Kir2.1 (Genepaint set ID: RB 77)

**Fig. 4:**
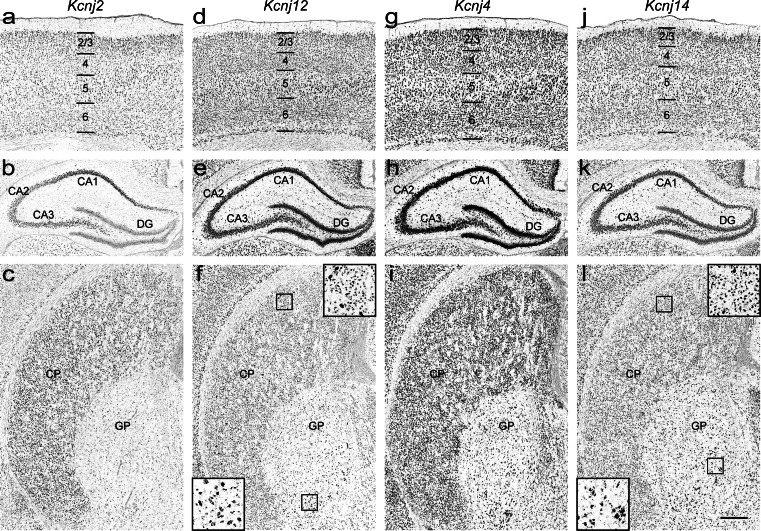
Expression patterns of classical *Kcnj* channel genes in the P14 rat brain. **a–c** *Kcnj2*, **d–f** *Kcnj12*, **g–i** *Kcnj4*, and **j–l** *Kcnj14* in the cerebral cortex (*top row*), hippocampus (*middle row*) and caudate putamen (*bottom row*). Insets represent high power views. *Abbreviations*: *1*-*6* cortical layers; *CA1 to 3* cornu ammonis, *CP* caudate putamen, *DG* dentate gyrus, *GP* globus pallidus. Scale bar = 200 μm

Weak expression of *Kcnj2* extends across the entire brain. However, there is elevation of signal in hippocampus (e.g. CA1, Fig. 4b), caudate putamen (Fig. 4c), mammillary nucleus, and anterodorsal and anteroventral thalamic nuclei. Furthermore, cortical layers 2/3 show more distinct expression (Fig. 4a), whereas signal is stronger in the medial plane and becomes weaker in the lateral plane. In previous studies, expression was not detected in the thalamus of adult rat by radioactive ISH (Karschin et al. 1996). However, it was shown (Karschin and Karschin 1997) that *Kcnj2* has pronounced expression in the thalamus, red nucleus, brainstem and cerebellum during development, and these structures were negative in the adult. The presence of *Kcnj2* mRNA in thalamic nuclei of the P14 rat brain, but not in the adult, could thus be reflecting transient developmental expression (Karschin and Karschin 1997). Of note, immunochemistry experiments in adult rat revealed elevated *Kcnj2* signal in cortex, hippocampus, anterodorsal and anteroventral thalamic nuclei (Pruss et al. 2005) and caudate putamen (Pruss et al. 2003).

#### *Kcnj12*/Kir2.2 (Genepaint set ID: RB 52)

*Kcnj12* mRNA is widely distributed in P14 rat brain with higher levels in the cerebral cortex, hippocampus (Fig. 4d and e), thalamus, medial habenula, pontine nucleus and the optic nerve layer of the superior colliculus. In the caudate putamen, scattered moderate expression was detected, but large cholinergic interneurons of the caudate putamen and many cells in globus pallidus were strongly labeled (Fig. 4f, insets). This pattern of expression is in broad agreement with previous studies (Karschin et al. 1996; Karschin and Karschin 1997; Pruss et al. 2003).

#### *Kcnj4*/Kir2.3 (Genepaint set ID: RB 102)

Highest *Kcnj4* mRNA levels were observed in the cerebral cortex, hippocampus, caudate putamen (Fig. 4g–i), piriform cortex, basolateral amygdaloid nucleus and reticular thalamic nucleus, thereby agreeing with previous studies (Karschin et al. 1996; Pruss et al. 2005) and adult mouse brain expression data (Lein et al. 2007). The remaining brain regions exhibit only weak expression.

#### *Kcnj14*/Kir2.4 (Genepaint set ID: RB 54)

*Kcnj14* is broadly expressed throughout the P14 rat brain but there is variance in expression strength. Only weak expression was seen in the hypothalamus and the choroid plexus, while expression was elevated in the substantia nigra as well as in the basolateral amygdaloid nucleus, red nucleus, and oculomotor nucleus. Cerebral cortex (more pronounced in layers 2/3), hippocampus (Fig. 4j and k) and thalamus showed moderate levels of *Kcnj14*. Furthermore, elevated signals were found in large cholinergic interneurons of the caudate putamen and in a population of cells in globus pallidus (Fig. 4l, insets). The expression patterns of *Kcnj14* in P14 rat brain differ from previous radioactive and non-radioactive ISH studies of adult rat (Topert et al. 1998), where this subunit was reported to be predominately expressed in motoneurons of cranial nerve motor nuclei at high levels, but was absent in other brain regions. Another study (Pruss et al. 2005) based on immunohistochemical and ISH experiments also reported strong *Kcnj14* expression in motoneurons, but found elevated levels of transcript and protein in rat neocortex, hippocampus, thalamus and oculomotor nucleus. Moreover, protein was also detected in large cholinergic interneurons of the caudate putamen (Pruss et al. 2003) thus agreeing with our results. The discrepancies between these findings could be due to differences in detection sensitivity and probe composition.

## G Protein-Gated *Kcnj* Channels (Kir3.x)

### *Kcnj3*/Kir3.1 (Genepaint set ID: RB 78)

**Fig. 5:**
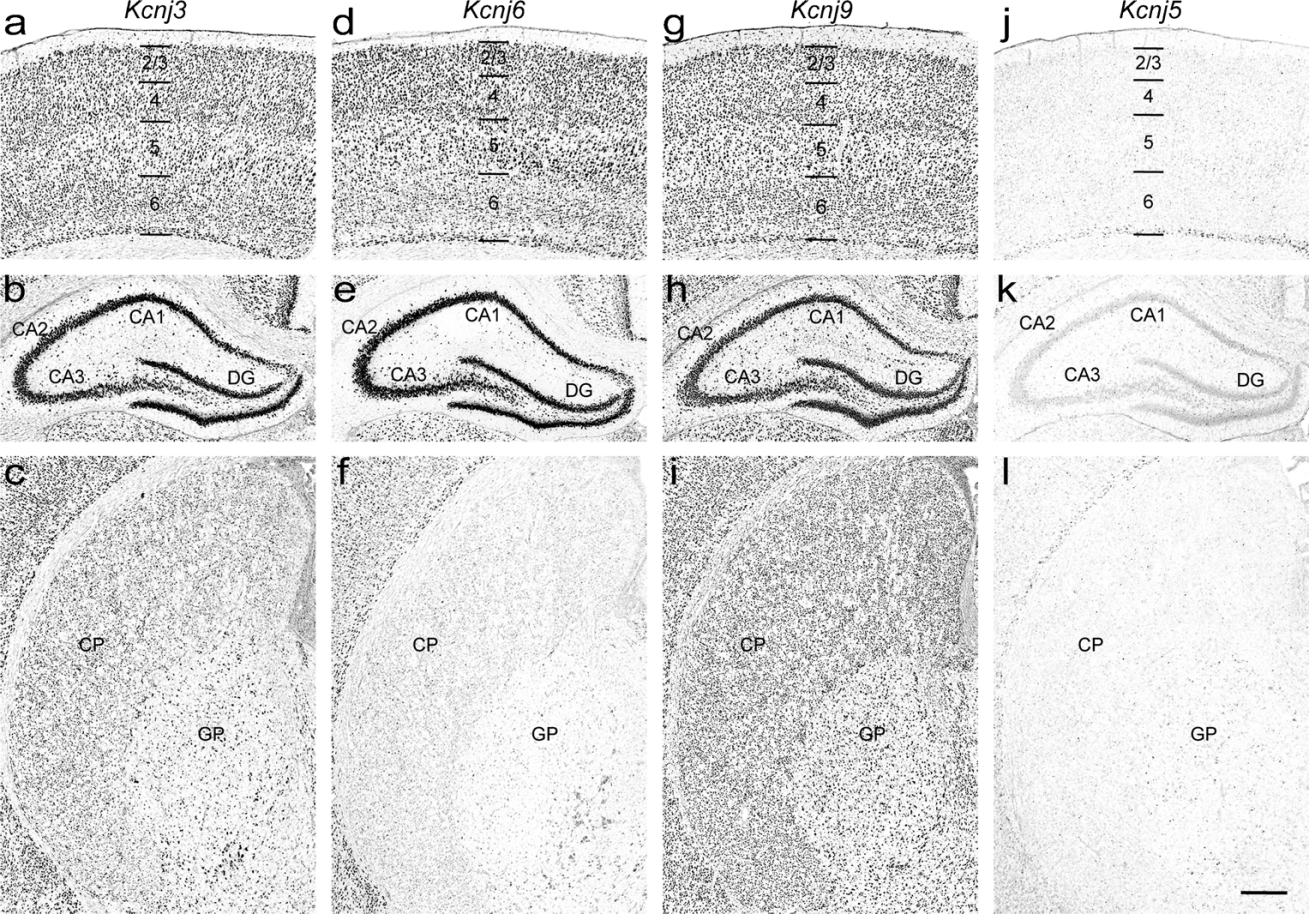
Expression patterns of G protein-gated *Kcnj* channel genes in the P14 rat brain. **a–c** *Kcnj3*, **d–f** *Kcnj6*, **g–i** *Kcnj9*, and **j–l** *Kcnj5* in the cerebral cortex (*top row*), hippocampus (*middle row*) and caudate putamen (*bottom row*). *Abbreviations*: see Fig. 4. Scale bar = 200 μm

Pronounced strong *Kcnj3* expression is seen in the cerebral cortex, hippocampus (Fig. 5a and b), thalamus, and the red and oculomotor nuclei. Caudate putamen (Fig. 5c), lateral septal nucleus and pontine nucleus show moderate expression levels. Most noteworthy, ventromedial hypothalamic nucleus and substantia nigra pars reticulata exhibit elevated signal, whereas the surrounding areas contain smaller amounts of transcript. These data largely agree with earlier reports and adult mouse brain expression data (Karschin et al. 1994; Karschin et al. 1996; Karschin and Karschin 1997; Chen et al. 1997; Lein et al. 2007; Saenz del Burgo et al. 2008).

### *Kcnj6*/Kir3.2 (Genepaint set ID: RB 103)

**Fig. 6:**
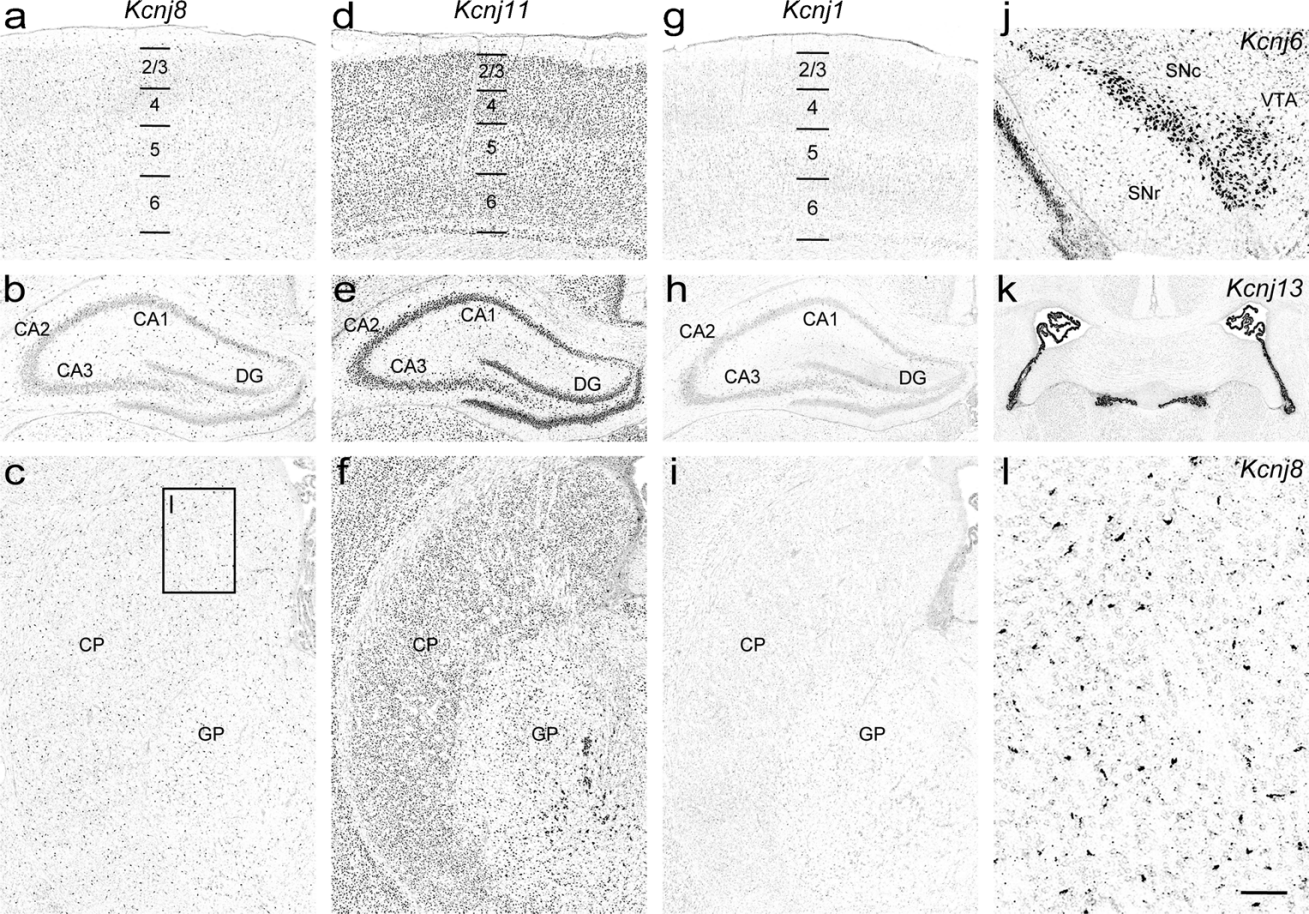
Differential expression of *Kcnj* channel genes in the P14 rat brain. ATP-sensitive K^+^ channels: **a–c** *Kcnj8* and **d–f** *Kcnj11*; and K^+^ transport channel: **g–i** *Kcnj1* in the cerebral cortex (*top row*), hippocampus (*middle row*) and caudate putamen (*bottom row*). **j** Strong regional expression of *Kcnj6* in the dopaminergic neurons of the substantia nigra pars compacta and ventral tegmental area. **k** *Kcnj13* in the choroid epithelium of the 3rd and the lateral ventricles. **l** Expression of the *Kcnj8* in cells likely to represent pericytes that are part of brain microvessels. *Abbreviations*: see Fig. 4 and additionally *SNc* substantia nigra pars compacta; *SNr* substantia nigra pars reticulata; *VTA* ventral tegmental area. Scale bars: *a*-*j*, 200 μm; *k*, 400 μm; *l*, 50 μm

Strong regional expression of *Kcnj6* was detected in the anterodorsal, parafascicular and lateral geniculate thalamic nuclei, optic layer of the superior colliculus, in the dopaminergic neurons of the substantia nigra pars compacta as well as in the ventral tegmental area (Fig. 6j). Furthermore, *Kcnj6* is significantly expressed in the hippocampus (Fig. 5e). Moderate levels were seen in the cerebral cortex (Fig. 5d), thalamus, lateral septal nucleus and pontine nucleus. In the remaining regions (e.g. caudate putamen (Fig. 5f), hypothalamus, midbrain and choroid plexus epithelium) only weak *Kcnj6* expression was found. Sites and strength of expression are in good agreement with earlier studies in rat and mouse brain (Karschin et al. 1996; Karschin and Karschin 1997; Murer et al. 1997; Chen et al. 1997; Thiery et al. 2003; Saenz del Burgo et al. 2008; Lein et al. 2007).

### *Kcnj9*/Kir3.3 (Genepaint set ID: RB 56)

The *Kcnj9* mRNA is the most ubiquitously expressed among this channel family. Its strength ranges from strong over medium to weak. Cerebral cortex, hippocampus (Fig. 5g and h), thalamus and pontine nucleus exhibit strong expression. Furthermore, moderate levels of expression were found in the caudate putamen (Fig. 5i), amygdala, and septum as well as in the hypothalamus and midbrain. Medial septal nucleus, basolateral amygdaloid nucleus, mammillary body and substantia nigra showed elevated signals. Only the choroid epithelium of the 3rd and lateral ventricles was weakly labeled. This pattern is consistent with previous reports and with the mouse brain expression data (Karschin et al. 1996; Karschin and Karschin 1997; Murer et al. 1997; Chen et al. 1997; Lein et al. 2007; Saenz del Burgo et al. 2008).

### *Kcnj5*/Kir3.4 (Genepaint set ID: RB 117)

Expression patterns of the *Kcnj5* mRNA are characterized by a scattered weak signal in almost all regions of the brain apart from the hippocampus (Fig. 5k) where ubiquitous weak expression signal is detected. Furthermore, cortical pyramidal neurons of layer 6 (Fig. 5j), parafascicular thalamic and ventromedial hypothalamic nuclei, and the optic nerve layer of the superior colliculus showed an elevated signal. The expression patterns for *Kcnj5* confirm earlier immunochemistry and ISH studies (Murer et al. 1997; Karschin and Karschin 1997; Wickman et al. 2000).

## ATP-Sensitive K^+^ Channels (Kir6.x)

### *Kcnj8*/Kir6.1 (Genepaint set ID: RB 104)

*Kcnj8* exhibits weak expression with scattered moderate levels of expression signal in distinct populations of cells throughout the brain (Fig. 6a–c) (Karschin et al. 1997; Zhou et al. 1999; Thomzig et al. 2001; Thomzig et al. 2005). It has recently been shown that *Kcnj8*, along with its auxiliary subunit *Abcc9* (ATP-binding cassette, subfamily C [CFTR/MRP], member 9), are novel markers for pericytes and the channel is specifically expressed in brain pericytes (Bondjers et al. 2006). It thus appears that the *Kcnj8*-positive elongated cells (Fig. 6l) often extending blood vessels are pericytes. In the adult mouse brain this signal is also present (Lein et al. 2007).

### *Kcnj11*/Kir6.2 (Genepaint set ID: RB 51)

*Kcnj11* mRNA is widely and differentially distributed throughout the rat brain (Fig. 6d–f). Moderate levels were detected in the cerebral cortex, hippocampus, thalamus and pons. While most of the remaining brain areas were weakly labelled, the subthalamic nucleus, the ventromedial hypothalamic nucleus and the substantia nigra showed elevated signal. The expression patterns we found confirm earlier studies (Karschin et al. 1997; Dunn-Meynell et al. 1998; Zhou et al. 2002; Thomzig et al. 2005).

## K^+^ Transport Channels (Kir1.1, Kir4.x, Kir5.1, Kir7.1)

### *Kcnj1*/Kir1.1 (Genepaint set ID: RB 100)

*Kcnj1* exhibits scattered weak expression across the entire P14 rat brain (Fig. 6g–i). Hence our data suggest a less regional pattern than had previously been reported (Kenna et al. 1994). Radioactive ISH revealed high levels of expression in the hippocampus and cortex but not in substantia nigra, ventromedial hypothalamus or caudate putamen. Note, however, two independent studies also demonstrated weak expression of *Kcnj1* in adult rat brain (Karschin et al. 1994; Wu et al. 2004), which is consistent with our results. Again, such apparent discrepancies between radioactive and non-radioactive ISH may simply be due to differences in detection sensitivity and probe composition.

### *Kcnj10*/Kir4.1 (Genepaint set ID: RB 50)

**Fig. 7:**
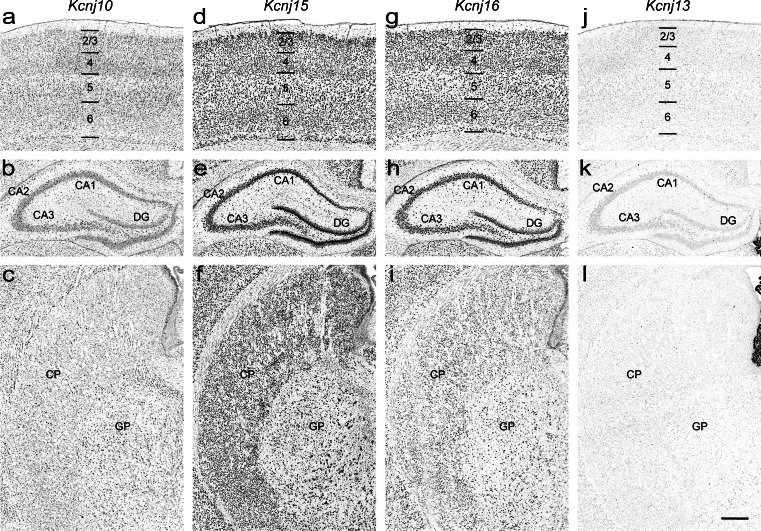
Expression patterns of K^+^ transport channels in the P14 rat brain. **a–c** *Kcnj10*, **d–f** *Kcnj15*, **g–i** *Kcnj16*, and **j–l** *Kcnj13* in the cerebral cortex (*top row*), hippocampus (*middle row*) and caudate putamen (*bottom row*). *Abbreviations*: see Fig. 4. Scale bar = 200 μm

*Kcnj10* shows weak expression accompanied by scattered moderate levels of signal in distinct population of cells throughout the brain (Fig.7a–c). This overall expression pattern is due to *Kcnj10* being expressed in astroglial cells (Takumi et al. 1995; Butt and Kalsi 2006), which are broadly distributed in the central nervous system. The expression patterns we describe have been previously reported (Poopalasundaram et al. 2000; Higashi et al. 2001; Ishii et al. 2003; Hibino et al. 2004).

### *Kcnj15*/Kir4.2 (Genepaint set ID: RB 55)

*Kcnj15* mRNA is widely distributed throughout all brain regions (Fig. 7d–f). Expression levels are generally moderate except for elevated signals in cerebral cortex (more pronounced in layers 2/3 and to some extent also 5 and 6, Fig. 7d), in the granular cell layer of the dentate gyrus (Fig. 7e) and in the substantia nigra. Our findings are consistent with previous reports for mouse (Thiery et al. 2003; Lein et al. 2007).

### *Kcnj16*/Kir5.1 (Genepaint set ID: RB 101)

Weak ubiquitous expression of *Kcnj16* is found in the majority of brain regions of the P14 rat (Fig. 7g–i). The cerebral cortex (Fig. 7g), hippocampus (Fig. 7h) and thalamus showed elevated signals. Both radioactive ISH and immunohistochemistry studies are in general agreement with our results (Derst et al. 2001; Ishii et al. 2003; Hibino et al. 2004; Wu et al. 2004).

### *Kcnj13*/Kir7.1 (Genepaint set ID: RB 53)

Strong regional expression of *Kcnj13* is restricted to the choroid plexus of the 3rd and lateral ventricles (Fig. 6k). This is consistent with the mouse expression data and the literature (Doring et al. 1998; Nakamura et al. 1999; Lein et al. 2007) in which no expression in other brain regions was detected (Fig. 7j–l).

**Fig. 8:**
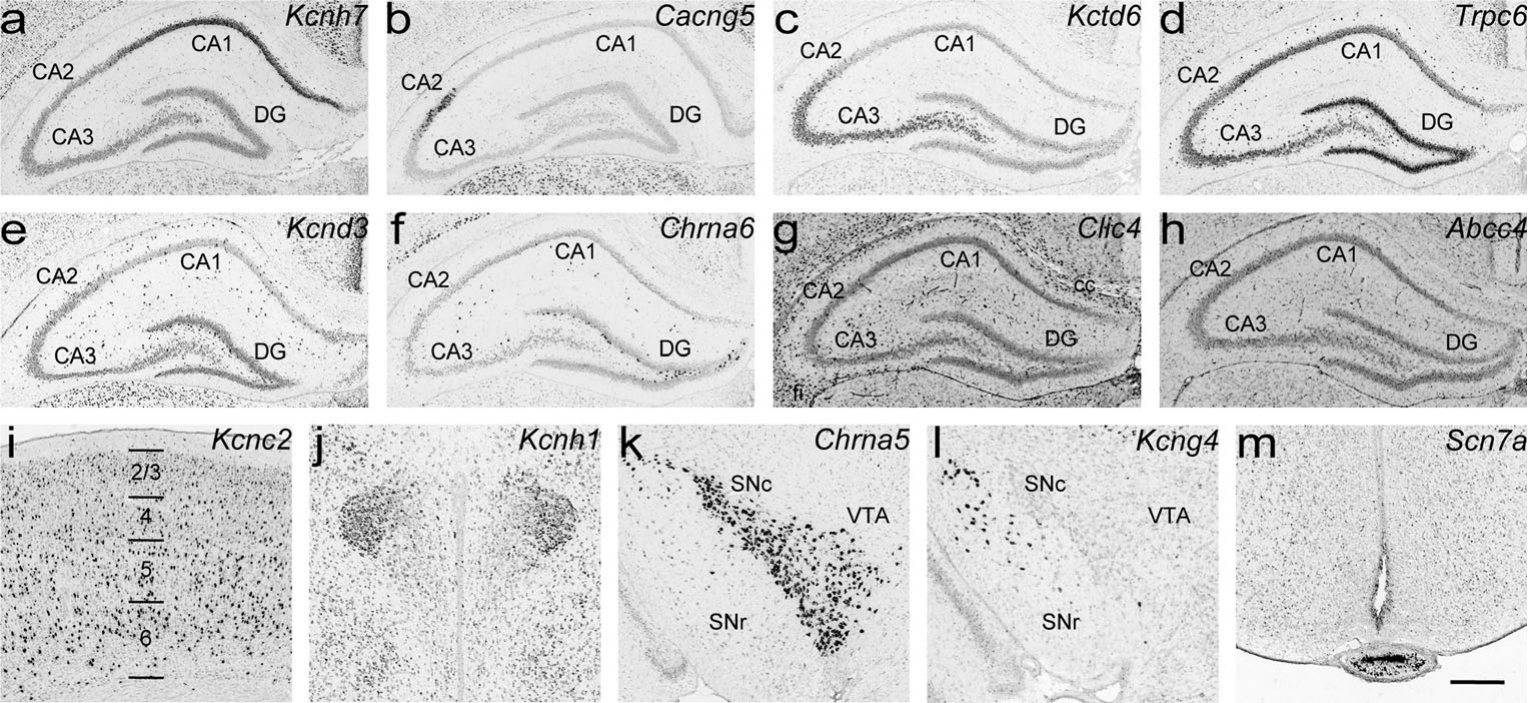
Differential expression of ion channel genes in the P14 rat brain. **a**–**d** Regionally enriched gene expression in the hippocampus: **a** *Kcnh7* in CA1, **b** *Cacng5* in CA2, **c** *Kctd6* in CA3, and **d** *Trpc6* in DG. **e**–**h** Heterogeneous expression in hippocampus: **e** *Kcnd3* in interneuron-like cell population, **f** *Chrna6* is enriched in a scattered cell population of the stratum radiatum and inner edge of the granular cell layer of dentate gyrus, **g** *Clic4* in oligodendrocytes, and **h** vascular cell-type expression of *Abcc4*. **i** *Kcnc2* in interneuron-like cell population in the cerebral cortex. **j** *Kcnh1* is regionally enriched in lateral magnocellular part of the PVH. **k**, **l** Differential channel gene expression in substantia nigra: **k** strong regional expression of *Chrna5* in pars compacta and **l** *Kcng4* in pars reticulata, respectively. **m** Strong regional expression of *Scn7a* in the median eminence. *Abbreviations*: see Fig. 4 and additionally *cc* corpus callosum; *fi* fimbria of hippocampus. Scale bar = 200 μm

Taken together, ion channels are broadly and differentially expressed in major brain regions (Fig. 8). Our study has identified numerous channel genes which are regionally enriched in the main subregions of the hippocampus (Fig. 8a–d) or in a particular nucleus (*Kcnh1* in lateral magnocellular part of the PVH, Fig. 8j). In substantia nigra, *Chrna5* and *Kcng4* are differentially expressed in pars compacta and pars reticulata, respectively (Fig. 8k–l). *Scn7a* is found to be strongly expressed in the median eminence and in the ventral part of the ependyma of the adjacent hypothalamic region (Fig. 8m).

Ion channels are heterogeneously expressed in distinct cell population throughout rat brain. Classical *Kcnj* (*Kcnj2*, *4*, *12* and *14*, Fig. 4) and G protein-gated *Kcnj* channels (*Kcnj3*, *5*, *6* and *9*, Fig. 5) are predominantly expressed in neurons (Koyrakh, 2005; Burgo, 2008; Murer, 1997). In contrast, *Kcnj10* and *Kcnj16* subunits (Fig. 7) are abundantly distributed in astroglial cells (Hibino et al. 2004), thus these genes may serve as novel candidates for astrocyte-specific markers. Channel genes are also detected in interneuron-like cell population at high levels (Fig. 8e, f and i). For example, *Chrna6* is strongly expressed in interneurons in CA1-3 stratum radiatum and inner edge of the granular cell layer of dentate gyrus (Fig. 8f). In addition, *Clic4* and *Abcc4* are enriched in oligodendrocytes and in blood vessels, respectively (Fig. 8g–h).

## Conclusions and Outlook

The objective of the present study was twofold. First, we systematically determined by robotic ISH the expression pattern of several hundred ion channels on standardized coronal sections of the P14 rat brain. These patterns have been made publicly available through Genepaint.org, an online database accessible at http://www.genepaint.org. Second, we used the same type of standardized coronal sections stained with cresyl violet to produce a deformable subdivision mesh coronal atlas of the P14 rat brain. We chose deformable subdivision meshes for atlas-based registration, because they have smooth region boundaries that can faithfully model the irregular surfaces typical for anatomical structures. Moreover, when combined with reproducible sectioning, particular mesh quadrilaterals within these subdivision regions generally coincide with the same anatomy across different brains.

To determine the ISH-based expression pattern of the several hundred members of the ion channel gene family and then compare these patterns, it is crucial that the expression patterns from the dozens of different brains required for this effort are placed into the same reference atlas. This goal can only be achieved if brains are reproducibly and consistently mounted and sectioned. Addressing this, we developed simple methods that do not require special equipment, and these methods yield comparable section planes from brains from different rats.

Approximately 350 individual genes encoding ion channels are present in the mammalian genome. We succeeded in generating ISH data sets for 320 channel genes of which 315 were expressed in the P14 brain. To examine the expression of the remaining ~30 genes, as well as for the over 90 genes that we knew from the ISH data were expressed in the brain, we undertook a quantitative PCR analysis. We found that for 8 channel genes we could detect expression in the brain by qPCR, for which we had not already been able to detect expression by ISH. In 17 cases channels were clearly not detectable in the brain by qPCR, although they were detected in one or more of the other tissues (thymus, lung and kidney) we examined. We conclude that our ISH-based expression patterns deposited at Genepaint.org are very close to capturing the rat channelome in full.

We focused on the *Kcnj* channel family as a test for the suitability of the developed methods and for a comparison of our expression results with those reported in scientific publications. To a large extent, our *Kcnj* expression patterns are consistent with those reported in the literature for the rat, but our data are generally more comprehensive and have higher resolution than e.g. radioactive ISH data. We also find consistency with expression data for the adult mouse, as shown in the Allen mouse brain atlas (http://mouse.brain-map.org/) (Lein et al. 2007).

In previous studies, double ISH experiments revealed co-expression of G protein-gated *Kcnj* channel subunits (*Kcnj3* and *Kcnj6*, *Kcnj3* and *Kcnj9*, and *Kcnj6* and *Kcnj9*) in majority of cells in the cerebral cortex, hippocampus and thalamus, with a less number of single-labeled cells (Saenz del Burgo et al. 2008). We have also found largely overlapping distributions of *Kcnj* channel subunits in distinct brain regions (Fig. 3). Note, however, that co-expression of ion channel subunits at mRNA and protein levels, even those from the same family and in the same cell, does not necessarily mean formation of heteromeric assemblies (Pessia et al. 2001; Konstas et al. 2003). Although, mRNA co-expression is necessary, it is not sufficient, to determine whether subunits co-assemble, and that can only be shown by methods capturing direct protein-protein interactions. In the cerebral cortex, both homomeric *Kcnj10* and heteromeric *Kcnj10*/*Kcnj16* channels are localized in the perisynaptic processes of brain astrocytes, while the heteromer is detected in the processes that attached to blood vessels (Hibino et al. 2004). Channel subunits may be transported outside the neuronal cell body, to distinct sites on axon and dendrites, whereas mRNA may be present on the cell membrane, and protein could thus reside in completely different brain regions than the mRNA (Murer et al. 1997; Karschin et al. 1997; Watakabe 2009).

Our first use of the subdivision atlas was the annotation and semi-quantitative comparison of the expression patterns of the *Kcnj* functional channels. In the future, the expression data for the remaining channel genes will also be placed into the subdivision mesh framework. This will allow analysis across channel subfamilies, shed light on the diversity of the channelome expression patterns and make comparisons quantifiable and comparable. Already now, the atlas-based interrogation of our expression data allows for the generation of expression reports (Table 2) that help to identify interesting expression patterns and sites that can be explored in more depth by retrieving the high-resolution data deposited at Genepaint.org. Another application of a registered atlas is to perform semi-quantitative correlations that include functionally linked channels.

Recent microarray studies have determined a number of ion channels that display enriched expression across cortical areas of the rat brain irrespective of cortical layers (Stansberg et al. 2011). Our atlas-based annotation offers an orthogonal approach that can identify both cortical-layer- and cortical-area-enriched channel genes which can be functionally related (Carson et al. 2005b). Additionally, double-labeling ISH affords identification of cellular preferences (neurons, astro- or oligodendrocytes) of the ion channel genes expressed in the brain,

## Information Sharing Statement

All expression data presented in this article are available through the Genepaint.org (RRID:nif-0000-00009) and freely available for public.

## Electronic Supplementary Material

Below is the link to the electronic supplementary material.

ESM 1 (PDF 360 kb)

ESM 2 (PDF 4460 kb)

## References

[CR1] Abraham MR, Jahangir A, Alekseev AE, Terzic A (1999). Channelopathies of inwardly rectifying potassium channels. FASEB Journal.

[CR2] Andersen ED, Krasilnikoff PA, Overvad H (1971). Intermittent muscular weakness, extrasystoles, and multiple developmental anomalies. A new syndrome?. Acta Paediatrica Scandinavica.

[CR3] Ashcroft FM (2005). ATP-sensitive potassium channelopathies: focus on insulin secretion. Journal of Clinical Investigation.

[CR4] Bartter FC, Pronove P, Gill JR, Maccardle RC (1962). Hyperplasia of the juxtaglomerular complex with hyperaldosteronism and hypokalemic alkalosis. A new syndrome. American Journal of Medicine.

[CR5] Benarroch EE (2009). Potassium channels: brief overview and implications in epilepsy. Neurology.

[CR6] Bendahhou S, Donaldson MR, Plaster NM, Tristani-Firouzi M, Fu YH, Ptacek LJ (2003). Defective potassium channel Kir2.1 trafficking underlies Andersen-Tawil syndrome. Journal of Biological Chemistry.

[CR7] Bondjers C, He L, Takemoto M, Norlin J, Asker N, Hellstrom M (2006). Microarray analysis of blood microvessels from PDGF-B and PDGF-Rbeta mutant mice identifies novel markers for brain pericytes. FASEB Journal.

[CR8] Butt AM, Kalsi A (2006). Inwardly rectifying potassium channels (Kir) in central nervous system glia: a special role for Kir4.1 in glial functions. Journal of Cellular and Molecular Medicine.

[CR9] Carson J, Thaller C, Eichele G (2002). A transcriptome atlas of the mouse brain at cellular resolution. Current Opinion in Neurobiology.

[CR10] Carson J, Ju T, Thaller C, Warren J, Bello M, Kakadiaris I (2004). Automated characterization of gene expression patterns with an atlas of the mouse brain. Conference Proceedings IEEE Engineering in Medicine and Biology Society.

[CR11] Carson J, Eichele G, Chiu W (2005). A method for automated detection of gene expression required for the establishment of a digital transcriptome-wide gene expression atlas. Journal of Microscopy.

[CR12] Carson J, Ju T, Lu HC, Thaller C, Xu M, Pallas SL (2005). A digital atlas to characterize the mouse brain transcriptome. PLoS Computational Biology.

[CR13] Carson J, Ju T, Bello M, Thaller C, Warren J, Kakadiaris IA (2010). Automated pipeline for atlas-based annotation of gene expression patterns: application to postnatal day 7 mouse brain. Methods.

[CR14] Chen SC, Ehrhard P, Goldowitz D, Smeyne RJ (1997). Developmental expression of the GIRK family of inward rectifying potassium channels: implications for abnormalities in the weaver mutant mouse. Brain Research.

[CR15] Derst C, Karschin C, Wischmeyer E, Hirsch JR, Preisig-Muller R, Rajan S (2001). Genetic and functional linkage of Kir5.1 and Kir2.1 channel subunits. FEBS Letters.

[CR16] Donaldson MR, Jensen JL, Tristani-Firouzi M, Tawil R, Bendahhou S, Suarez WA (2003). PIP2 binding residues of Kir2.1 are common targets of mutations causing Andersen syndrome. Neurology.

[CR17] Doring F, Derst C, Wischmeyer E, Karschin C, Schneggenburger R, Daut J (1998). The epithelial inward rectifier channel Kir7.1 displays unusual K+ permeation properties. Journal of Neuroscience.

[CR18] Dunn-Meynell AA, Rawson NE, Levin BE (1998). Distribution and phenotype of neurons containing the ATP-sensitive K+ channel in rat brain. Brain Research.

[CR19] Eichele G, Diez-Roux G (2011). High-throughput analysis of gene expression on tissue sections by in situ hybridization. Methods.

[CR20] Fuller L, Dailey ME (2007). Preparation of rodent hippocampal slice cultures. CSH Protocol.

[CR21] Gosset P, Ghezala GA, Korn B, Yaspo ML, Poutska A, Lehrach H (1997). A new inward rectifier potassium channel gene (KCNJ15) localized on chromosome 21 in the Down syndrome chromosome region 1 (DCR1). Genomics.

[CR22] Hibino H, Fujita A, Iwai K, Yamada M, Kurachi Y (2004). Differential assembly of inwardly rectifying K+ channel subunits, Kir4.1 and Kir5.1, in brain astrocytes. Journal of Biological Chemistry.

[CR23] Hibino H, Inanobe A, Furutani K, Murakami S, Findlay I, Kurachi Y (2010). Inwardly rectifying potassium channels: their structure, function, and physiological roles. Physiological Reviews.

[CR24] Higashi K, Fujita A, Inanobe A, Tanemoto M, Doi K, Kubo T (2001). An inwardly rectifying K(+) channel, Kir4.1, expressed in astrocytes surrounds synapses and blood vessels in brain. American Journal of Physiology - Cellular Physiology.

[CR25] Ho K, Nichols CG, Lederer WJ, Lytton J, Vassilev PM, Kanazirska MV (1993). Cloning and expression of an inwardly rectifying ATP-regulated potassium channel. Nature.

[CR26] Ishihara K, Yamamoto T, Kubo Y (2009). Heteromeric assembly of inward rectifier channel subunit Kir2.1 with Kir3.1 and with Kir3.4. Biochemical and Biophysical Research Communications.

[CR27] Ishii M, Fujita A, Iwai K, Kusaka S, Higashi K, Inanobe A (2003). Differential expression and distribution of Kir5.1 and Kir4.1 inwardly rectifying K+ channels in retina. American Journal of Physiology - Cellular Physiology.

[CR28] Ju T, Carson J, Liu L, Warren J, Bello M, Kakadiaris I (2010). Subdivision meshes for organizing spatial biomedical data. Methods.

[CR29] Judge SI, Smith PJ, Stewart PE, Bever CT (2007). Potassium channel blockers and openers as CNS neurologic therapeutic agents. Recent Patents on CNS Drug Discovery.

[CR30] Karschin C, Karschin A (1997). Ontogeny of gene expression of Kir channel subunits in the rat. Molecular and Cellular Neuroscience.

[CR31] Karschin C, Schreibmayer W, Dascal N, Lester H, Davidson N, Karschin A (1994). Distribution and localization of a G protein-coupled inwardly rectifying K+ channel in the rat. FEBS Letters.

[CR32] Karschin C, Dissmann E, Stuhmer W, Karschin A (1996). IRK(1-3) and GIRK(1-4) inwardly rectifying K+ channel mRNAs are differentially expressed in the adult rat brain. Journal of Neuroscience.

[CR33] Karschin C, Ecke C, Ashcroft FM, Karschin A (1997). Overlapping distribution of K(ATP) channel-forming Kir6.2 subunit and the sulfonylurea receptor SUR1 in rodent brain. FEBS Letters.

[CR34] Kenna S, Roper J, Ho K, Hebert S, Ashcroft SJ, Ashcroft FM (1994). Differential expression of the inwardly-rectifying K-channel ROMK1 in rat brain. Brain Research. Molecular Brain Research.

[CR35] Konstas AA, Korbmacher C, Tucker SJ (2003). Identification of domains that control the heteromeric assembly of Kir5.1/Kir4.0 potassium channels. American Journal of Physiology - Cellular Physiology.

[CR36] Krapivinsky G, Gordon EA, Wickman K, Velimirovic B, Krapivinsky L, Clapham DE (1995). The G-protein-gated atrial K+ channel IKACh is a heteromultimer of two inwardly rectifying K(+)-channel proteins. Nature.

[CR37] Kubo Y, Reuveny E, Slesinger PA, Jan YN, Jan LY (1993). Primary structure and functional expression of a rat G-protein-coupled muscarinic potassium channel. Nature.

[CR38] Kubo Y, Adelman JP, Clapham DE, Jan LY, Karschin A, Kurachi Y (2005). International Union of Pharmacology. LIV. Nomenclature and molecular relationships of inwardly rectifying potassium channels. Pharmacological Reviews.

[CR39] Lein ES, Hawrylycz MJ, Ao N, Ayres M, Bensinger A, Bernard A (2007). Genome-wide atlas of gene expression in the adult mouse brain. Nature.

[CR40] Lu Z (2004). Mechanism of rectification in inward-rectifier K+ channels. Annual Review of Physiology.

[CR41] Miki T, Liss B, Minami K, Shiuchi T, Saraya A, Kashima Y (2001). ATP-sensitive K+ channels in the hypothalamus are essential for the maintenance of glucose homeostasis. Nature Neuroscience.

[CR42] Murer G, Adelbrecht C, Lauritzen I, Lesage F, Lazdunski M, Agid Y (1997). An immunocytochemical study on the distribution of two G-protein-gated inward rectifier potassium channels (GIRK2 and GIRK4) in the adult rat brain. Neuroscience.

[CR43] Naesens M, Steels P, Verberckmoes R, Vanrenterghem Y, Kuypers D (2004). Bartter’s and Gitelman’s syndromes: from gene to clinic. Nephron. Physiology.

[CR44] Nakamura N, Suzuki Y, Sakuta H, Ookata K, Kawahara K, Hirose S (1999). Inwardly rectifying K+ channel Kir7.1 is highly expressed in thyroid follicular cells, intestinal epithelial cells and choroid plexus epithelial cells: implication for a functional coupling with Na+, K+-ATPase. Biochemical Journal.

[CR45] Neusch C, Weishaupt JH, Bahr M (2003). Kir channels in the CNS: emerging new roles and implications for neurological diseases. Cell and Tissue Research.

[CR46] Nichols CG, Lopatin AN (1997). Inward rectifier potassium channels. Annual Review of Physiology.

[CR47] Nishida M, MacKinnon R (2002). Structural basis of inward rectification: cytoplasmic pore of the G protein-gated inward rectifier GIRK1 at 1.8 A resolution. Cell.

[CR48] Patil N, Cox DR, Bhat D, Faham M, Myers RM, Peterson AS (1995). A potassium channel mutation in weaver mice implicates membrane excitability in granule cell differentiation. Nature Genetics.

[CR49] Paxinos G, Watson C (2013). The Rat Brain in Stereotaxic Coordinates.

[CR50] Pessia M, Imbrici P, D’Adamo MC, Salvatore L, Tucker SJ (2001). Differential pH sensitivity of Kir4.1 and Kir4.2 potassium channels and their modulation by heteropolymerisation with Kir5.1. Journal of Physiology.

[CR51] Poopalasundaram S, Knott C, Shamotienko OG, Foran PG, Dolly JO, Ghiani CA (2000). Glial heterogeneity in expression of the inwardly rectifying K(+) channel, Kir4.1, in adult rat CNS. Glia.

[CR52] Preisig-Muller R, Schlichthorl G, Goerge T, Heinen S, Bruggemann A, Rajan S (2002). Heteromerization of Kir2.x potassium channels contributes to the phenotype of Andersen’s syndrome. Proceedings of the National Academy of Sciences of the United States of America.

[CR53] Pruss H, Wenzel M, Eulitz D, Thomzig A, Karschin A, Veh RW (2003). Kir2 potassium channels in rat striatum are strategically localized to control basal ganglia function. Brain Research. Molecular Brain Research.

[CR54] Pruss H, Derst C, Lommel R, Veh RW (2005). Differential distribution of individual subunits of strongly inwardly rectifying potassium channels (Kir2 family) in rat brain. Brain Research. Molecular Brain Research.

[CR55] Reimann F, Ashcroft FM (1999). Inwardly rectifying potassium channels. Current Opinion in Cell Biology.

[CR56] Reymond A, Marigo V, Yaylaoglu MB, Leoni A, Ucla C, Scamuffa N (2002). Human chromosome 21 gene expression atlas in the mouse. Nature.

[CR57] Saenz del Burgo L, Cortes R, Mengod G, Zarate J, Echevarria E, Salles J (2008). Distribution and neurochemical characterization of neurons expressing GIRK channels in the rat brain. Journal of Comparative Neurology.

[CR58] Schram G, Melnyk P, Pourrier M, Wang Z, Nattel S (2002). Kir2.4 and Kir2.1 K(+) channel subunits co-assemble: a potential new contributor to inward rectifier current heterogeneity. Journal of Physiology.

[CR59] Shimomura K (2009). The K(ATP) channel and neonatal diabetes. Endocrine Journal.

[CR60] Stansberg C, Ersland KM, van der Valk P, Steen VM (2011). Gene expression in the rat brain: high similarity but unique differences between frontomedial-, temporal- and occipital cortex. BMC Neuroscience.

[CR61] Sun XL, Hu G (2010). ATP-sensitive potassium channels: a promising target for protecting neurovascular unit function in stroke. Clinical and Experimental Pharmacology and Physiology.

[CR62] Takumi T, Ishii T, Horio Y, Morishige K, Takahashi N, Yamada M (1995). A novel ATP-dependent inward rectifier potassium channel expressed predominantly in glial cells. Journal of Biological Chemistry.

[CR63] Thiery, E., Thomas, S., Vacher, S., Delezoide, A. L., Delabar, J. M., & Creau, N. (2003). Chromosome 21 KIR channels in brain development. *J Neural Transm Suppl* (67):105-115. 10.1007/978-3-7091-6721-2_9 15068243

[CR64] Thomzig A, Wenzel M, Karschin C, Eaton MJ, Skatchkov SN, Karschin A (2001). Kir6.1 is the principal pore-forming subunit of astrocyte but not neuronal plasma membrane K-ATP channels. Molecular and Cellular Neuroscience.

[CR65] Thomzig A, Laube G, Pruss H, Veh RW (2005). Pore-forming subunits of K-ATP channels, Kir6.1 and Kir6.2, display prominent differences in regional and cellular distribution in the rat brain. Journal of Comparative Neurology.

[CR66] Topert C, Doring F, Wischmeyer E, Karschin C, Brockhaus J, Ballanyi K (1998). Kir2.4: a novel K+ inward rectifier channel associated with motoneurons of cranial nerve nuclei. Journal of Neuroscience.

[CR67] Visel A, Thaller C, Eichele G (2004). GenePaint.org: an atlas of gene expression patterns in the mouse embryo. Nucleic Acids Research.

[CR68] Warren J, Weimer H (2002). Subdivision methods for geometric design: a constructive approach.

[CR69] Watakabe A (2009). Comparative molecular neuroanatomy of mammalian neocortex: what can gene expression tell us about areas and layers?. Development, Growth & Differentiation.

[CR70] Wickman K, Karschin C, Karschin A, Picciotto MR, Clapham DE (2000). Brain localization and behavioral impact of the G-protein-gated K+ channel subunit GIRK4. Journal of Neuroscience.

[CR71] Wu J, Xu H, Shen W, Jiang C (2004). Expression and coexpression of CO2-sensitive Kir channels in brainstem neurons of rats. Journal of Membrane Biology.

[CR72] Yaylaoglu MB, Titmus A, Visel A, Alvarez-Bolado G, Thaller C, Eichele G (2005). Comprehensive expression atlas of fibroblast growth factors and their receptors generated by a novel robotic in situ hybridization platform. Developmental Dynamics.

[CR73] Yu FH, Catterall WA (2004). The VGL-chanome: a protein superfamily specialized for electrical signaling and ionic homeostasis. Science’s STKE.

[CR74] Zhou M, Tanaka O, Sekiguchi M, Sakabe K, Anzai M, Izumida I (1999). Localization of the ATP-sensitive potassium channel subunit (Kir6. 1/uK(ATP)-1) in rat brain. Brain Research. Molecular Brain Research.

[CR75] Zhou M, Tanaka O, Suzuki M, Sekiguchi M, Takata K, Kawahara K (2002). Localization of pore-forming subunit of the ATP-sensitive K(+)-channel, Kir6.2, in rat brain neurons and glial cells. Brain Research. Molecular Brain Research.

