# Supplementary material for: A Digital Atlas of Ion Channel Expression Patterns in the Two-Week-Old Rat Brain
Source: Neuroinformatics. 2014 Oct 7;13(1):111–25. doi: 10.1007/s12021-014-9247-0 (PMC4303740; doi:10.1007/s12021-014-9247-0)
Supplement: Supplementary file 1 — (PDF 360 kb) [file 12021_2014_9247_MOESM1_ESM.pdf]

A Digital Atlas of Ion Channel Expression Patterns in the Two-Week-Old Rat Brain

Volodymyr Shcherbatyy[1], James Carson[2], Murat Yaylaoglu[1], Katharina Jäckle[1], Frauke Grabbe[1], Maren Brockmeyer [1], Halenur Yavuz [1], and Gregor Eichele[1*]

[1] *Department of Genes and Behavior, Max Plank Institute for Biophysical Chemistry, Göttingen, Germany*
[2] *Life Sciences Computing, Texas Advanced Computing Center, Austin, TX, USA*

[*] Correspondence to: Gregor Eichele, Department of Genes and Behavior, Max Planck Institute for Biophysical Chemistry, Am Fassberg 11, 37077 Göttingen, Germany
E-mail address: gregor.eichele@mpibpc.mpg.de

Online Resource 1

ESM_1. List of ion channels examined in the P14 rat brain.

ISH data are viewable using the entry interface of Genepaint.org (http://www.genepaint.org/) by entering the gene symbol or Genepaint set ID (a database internal ID). In the Genepaint.org database, images of individual sections can be viewed at full resolution by using the virtual microscope tools. Additionally, the full template sequence, specimen properties, gene accession number and Entrez Gene ID can be retrieved and images can be downloaded as described in the Manual of Genepaint under “Zoom Viewer” on the Genepaint.org home page [see also (Geffers et al. 2012)].

Supplemental references

Geffers, L., Herrmann, B., & Eichele, G. (2012). Web-based digital gene expression atlases for the mouse. *Mamm Genome, 23*(9-10), 525-538, doi:10.1007/s00335-012-9413-3.

ESM_1. List of ion channels examined in the P14 rat brain

Rat Gene Symbol	Gene Name	Genepaint Set Id	qPCR Analysis					
			Tissue				Primer	
			Brain	Thymus	Lung	Kidney	Forward	Reverse
**Calcium Voltage-Gated Channels**								
Cacna1a	Calcium channel voltage-dependent P/Q-type alpha-1A subunit	RB43						
Cacna1b	Calcium channel voltage-dependent N-type alpha-1B subunit	RB44						
Cacna1c	Calcium channel voltage-dependent L-type alpha-1C subunit	RB45						
Cacna1d	Calcium channel voltage-dependent L-type alpha-1D subunit	RB46						
Cacna1e	Calcium channel voltage-dependent R-type alpha-1E subunit	RB47						
Cacna1f	Calcium channel voltage-dependent L-type alpha-1F subunit	RB48						
Cacna1g	Calcium channel voltage-dependent T-type alpha-1G subunit	RB49						
Cacna1h	Calcium channel voltage-dependent T-type alpha-1H subunit	RB57						
Cacna1i	Calcium channel voltage-dependent T-type alpha-1I subunit	RB58						
Cacna1s	Calcium channel voltage-dependent L-type alpha-1S subunit	RB59						
Cacna2d1	Calcium channel voltage-dependent alpha 2/delta subunit 1	RB60						
Cacna2d2	Calcium channel voltage-dependent alpha 2/delta subunit 2	RB61						
Cacna2d3	Calcium channel voltage-dependent alpha 2/delta subunit 3	RB62						
Cacna2d4	Calcium channel voltage-dependent alpha 2/delta subunit 4	RB63						
Cacnb1	Calcium channel voltage-dependent beta-1 subunit	RB64						
Cacnb2	Calcium channel voltage-dependent beta-2 subunit	RB65						
Cacnb3	Calcium channel voltage-dependent beta-3 subunit	RB66						
Cacnb4	Calcium channel voltage-dependent beta-4 subunit	RB67						
Cacng1	Calcium channel voltage-dependent gamma-1 subunit		ND	ND	ND	ND	gcgtgaccctcttcttcatc	gtctgggttgtgtccatgtg
Cacng2	Calcium channel voltage-dependent gamma-2 subunit	RB68						
Cacng3	Calcium channel voltage-dependent gamma-3 subunit	RB69						
Cacng4	Calcium channel voltage-dependent gamma-4 subunit	RB70						
Cacng5	Calcium channel voltage-dependent gamma-5 subunit	RB71						

D: Detected (green); ND: Not Detected (yellow)

ESM_1. List of ion channels examined in the P14 rat brain

Rat Gene Symbol	Gene Name	Genepaint Set Id	qPCR Analysis					
			Tissue				Primer	
			Brain	Thymus	Lung	Kidney	Forward	Reverse
Cacng6	Calcium channel voltage-dependent gamma-6 subunit	RB72						
Cacng7	Calcium channel voltage-dependent gamma-7 subunit	RB73						
Cacng8	Calcium channel voltage-dependent gamma-8 subunit	RB74						
**Cyclic Nucleotide Gated Channels**								
Cnga1	Cyclic-nucleotide-gated cation channel alpha 1	RB130	D	D	D	D	cgtgcagctcattttctgac	ggtcctcgccttctgtatga
Cnga2	Cyclic-nucleotide-gated cation channel alpha 2		ND	ND	D	ND	actgtgacctcaggactctg	atctttgccattggccttga
Cnga3	Cyclic-nucleotide-gated cation channel alpha 3		ND	ND	ND	ND	ggagaccggagaactggctg	gctcagctccatgaaaacga
Cnga4	Cyclic-nucleotide-gated cation channel alpha 4	RB131	D	ND	ND	ND	attggtctatagatgcctag	cctcaagagtcccagtgcat
Cngb1	Cyclic-nucleotide-gated cation channel beta 1		ND	ND	ND	ND	aaggagaggatgtggctgag	ccttctccatgcccttccag
Cngb3	Cyclic-nucleotide-gated cation channel beta 3		ND	ND	D	D	ccaaacaccagacaacagga	actccgaagaagatgtagag
Hcn1	Potassium hyperpolarization-activated cyclic nucleotide-gated channel 1	RB123						
Hcn2	Potassium hyperpolarization-activated cyclic nucleotide-gated channel 2	RB319						
Hcn3	Potassium hyperpolarization-activated cyclic nucleotide-gated channel 3	RB334						
Hcn4	Potassium hyperpolarization-activated cyclic nucleotide-gated channel 4	RB340						
**Potassium Voltage-Gated Channels**								
Kcna1	Potassium voltage-gated channel subfamily A member 1	RB86						
Kcna2	Potassium voltage-gated channel subfamily A member 2	RB87						
Kcna3	Potassium voltage-gated channel subfamily A member 3	RB88						
Kcna4	Potassium voltage-gated channel subfamily A member 4	RB89						
Kcna5	Potassium voltage-gated channel subfamily A member 5	RB90						
Kcna6	Potassium voltage-gated channel subfamily A member 6	RB91						
Kcna7	Potassium voltage-gated channel subfamily A member 7	RB92						
Kcna10	Potassium voltage-gated channel subfamily A member 10	RB93						
Kcnab1	Potassium voltage-gated channe beta-1 subunit	RB94						

D: Detected (green); ND: Not Detected (yellow)

ESM_1. List of ion channels examined in the P14 rat brain

Rat Gene Symbol	Gene Name	Genepaint Set Id	qPCR Analysis					
			Tissue				Primer	
			Brain	Thymus	Lung	Kidney	Forward	Reverse
Kcnab2	Potassium voltage-gated channe beta-2 subunit	RB95						
Kcnab3	Potassium voltage-gated channe beta-3 subunit	RB96						
Kcnb1	Potassium voltage-gated channel subfamily B member 1	RB97						
Kcnb2	Potassium voltage-gated channel subfamily B member 2	RB98						
Kcnc1	Potassium voltage-gated channel subfamily C member 1	RB99						
Kcnc2	Potassium voltage-gated channel subfamily C member 2	RB79						
Kcnc3	Potassium voltage-gated channel subfamily C member 3	RB80						
Kcnc4	Potassium voltage-gated channel subfamily C member 4	RB175						
Kcnd1	Potassium voltage-gated channel subfamily D member 1	RB82						
Kcnd2	Potassium voltage-gated channel subfamily D member 2	RB83						
Kcnd3	Potassium voltage-gated channel subfamily D member 3	RB311						
Kcne1	Potassium voltage-gated channel subfamily E member 1	RB85						
Kcne1l	KCNE1-like	RB105						
Kcne2	Potassium voltage-gated channel subfamily E member 2	RB106						
Kcne3	Potassium voltage-gated channel subfamily E member 3	RB107						
Kcne4	Potassium voltage-gated channel subfamily E member 4	RB108	D	D	D	D	cagccgtttaagagttccac	tgtgctattcagaggctcca
Kcnf1	Potassium voltage-gated channel subfamily F member 1	RB109						
Kcng1	Potassium voltage-gated channel subfamily G member 1	RB110						
Kcng2	Potassium voltage-gated channel subfamily G member 2	RB111						
Kcng3	Potassium voltage-gated channel subfamily G member 3	RB335						
Kcng4	Potassium voltage-gated channel subfamily G member 4	RB152						
Kcnh1	Potassium voltage-gated channel subfamily H member 1	RB336						
Kcnh2	Potassium voltage-gated channel subfamily H member 2	RB337						
Kcnh3	Potassium voltage-gated channel subfamily H member 3	RB338						

D: Detected (green); ND: Not Detected (yellow)

ESM_1. List of ion channels examined in the P14 rat brain

Rat Gene Symbol	Gene Name	Genepaint Set Id	qPCR Analysis					
			Tissue				Primer	
			Brain	Thymus	Lung	Kidney	Forward	Reverse
Kcnh4	Potassium voltage-gated channel subfamily H member 4	RB339						
Kcnh5	Potassium voltage-gated channel subfamily H member 5	RB341						
Kcnh6	Potassium voltage-gated channel subfamily H member 6	RB342						
Kcnh7	Potassium voltage-gated channel subfamily H member 7	RB343						
Kcnh8	Potassium voltage-gated channel subfamily H member 8	RB344						
Kcnq1	Potassium voltage-gated channel subfamily KQT member 1	RB345						
Kcnq2	Potassium voltage-gated channel subfamily KQT member 2	RB346						
Kcnq3	Potassium voltage-gated channel subfamily KQT member 3	RB347						
Kcnq4	Potassium voltage-gated channel subfamily KQT member 4	RB348						
Kcnq5	Potassium voltage-gated channel subfamily KQT member 5	RB176						
Kcnrg	Potassium channels regulator	RB207						
Kcns1	Potassium voltage-gated channel subfamily S member 1	RB177						
Kcns2	Potassium voltage-gated channel subfamily S member 2	RB178						
Kcns3	Potassium voltage-gated channel subfamily S member 3	RB179						
Kcnv1	Potassium voltage-gated channel subfamily V member 1	RB180						
Kcnv2	Potassium voltage-gated channel subfamily V member 2	RB181						

Inwardly-Rectifying Potassium Channels

Kcnj1	Potassium inwardly-rectifying channel subfamily J member 1	RB100						
Kcnj2	Potassium inwardly-rectifying channel subfamily J member 2	RB77						
Kcnj3	Potassium inwardly-rectifying channel subfamily J member 3	RB78						
Kcnj4	Potassium inwardly-rectifying channel subfamily J member 4	RB102						
Kcnj5	Potassium inwardly-rectifying channel subfamily J member 5	RB117						
Kcnj6	Potassium inwardly-rectifying channel subfamily J member 6	RB103						
Kcnj8	Potassium inwardly-rectifying channel subfamily J member 8	RB104						

D: Detected (green); ND: Not Detected (yellow)

ESM_1. List of ion channels examined in the P14 rat brain

Rat Gene Symbol	Gene Name	Genepaint Set Id	qPCR Analysis					
			Tissue				Primer	
			Brain	Thymus	Lung	Kidney	Forward	Reverse
Kcnj9	Potassium inwardly-rectifying channel subfamily J member 9	RB56						
Kcnj10	Potassium inwardly-rectifying channel subfamily J member 10	RB50						
Kcnj11	Potassium inwardly-rectifying channel subfamily J member 11	RB51						
Kcnj12	Potassium inwardly-rectifying channel subfamily J member 12	RB52						
Kcnj13	Potassium inwardly-rectifying channel subfamily J member 13	RB53	D	ND	D	D	cagctgcgttctccttctct	ggtttgccatctttgtgagc
Kcnj14	Potassium inwardly-rectifying channel subfamily J member 14	RB54						
Kcnj15	Potassium inwardly-rectifying channel subfamily J member 15	RB55						
Kcnj16	Potassium inwardly-rectifying channel subfamily J member 16	RB101						
**Two-P Potassium Channels**								
Kcnk1	Potassium channel subfamily K member 1	RB124						
Kcnk2	Potassium channel subfamily K member 2	RB268						
Kcnk3	Potassium channel subfamily K member 3	RB125						
Kcnk4	Potassium channel subfamily K member 4	RB126						
Kcnk5	Potassium channel subfamily K member 5	RB127						
Kcnk6	Potassium channel subfamily K member 6	RB128						
Kcnk7	Potassium channel subfamily K member 7	RB129						
Kcnk9	Potassium channel subfamily K member 9	RB305						
Kcnk10	Potassium channel subfamily K member 10	RB267						
Kcnk12	Potassium channel subfamily K member 12	RB296						
Kcnk13	Potassium channel subfamily K member 13	RB297						
Kcnk15	Potassium channel subfamily K member 15	RB298						
Kcnk16	Potassium channel subfamily K member 16	RB299						
Kcnk18	Potassium channel subfamily K member 18		ND	ND	ND	ND	gtgggtgctgctctcttctc	ttcaaccactgtcaggttgc
**Calcium-Activated Potassium Channels**								

D: Detected (green); ND: Not Detected (yellow)

ESM_1. List of ion channels examined in the P14 rat brain

Rat Gene Symbol	Gene Name	Genepaint Set Id	qPCR Analysis					
			Tissue				Primer	
			Brain	Thymus	Lung	Kidney	Forward	Reverse
Kcnma1	Potassium calcium-activated channel subfamily M alpha member 1	RB313						
Kcnmb1	Potassium calcium-activated channel subfamily M beta member 1	RB314	D	D	D	D	actggagaccaaacttcctg	atggccaccggaggcaacag
Kcnmb2	Potassium calcium-activated channel subfamily M beta member 2	RB138						
Kcnmb3	Potassium calcium-activated channel subfamily M beta member 3		ND	ND	ND	ND	actttgccttcacctgtgag	agatgatccctgtctccgtg
Kcnmb4	Potassium calcium-activated channel subfamily M beta member 4	RB315						
Kcnn1	Potassium intermediate/small conductance calcium-activated channel subfamily N, member 1	RB316						
Kcnn2	Potassium intermediate/small conductance calcium-activated channel subfamily N, member 2	RB317						
Kcnn3	Potassium intermediate/small conductance calcium-activated channel subfamily N, member 3	RB318						
Kcnn4	Potassium intermediate/small conductance calcium-activated channel protein 4	RB351						
Kcnt1	Potassium channel subfamily T member 1							
Kcnt2	Potassium channel subfamily T member 2	RB132	D	D	D	D	ggatcagaacgtatgccaga	gaggttgagttgcggtgaat
Kcnu1	Potassium channel subfamily U member 1		ND	D	ND	ND	gtcaaggggccaagaggact	atgcgctgacgaaatactcc

Sodium Voltage-Gated Channels

Scn1a	Sodium channel voltage-gated type I alpha subunit	RB182						
Scn1b	Sodium channel voltage-gated type I beta subunit	RB183						
Scn2a1	Sodium channel voltage-gated type II alpha subunit	RB406	D	D	D	D	aacctgcactggagactgct	actgaccgtgccatcttttc
Scn2b	Sodium channel voltage-gated type II beta subunit	RB357						
Scn3a	Sodium channel voltage-gated type III alpha subunit	RB184						
Scn3b	Sodium channel voltage-gated type III beta subunit	RB185						
Scn4a	Sodium channel voltage-gated type IV alpha subunit	RB186						
Scn4b	Sodium channel voltage-gated type IV beta subunit	RB307						
Scn5a	Sodium channel voltage-gated type V alpha subunit	RB300						
Scn7a	Sodium channel voltage-gated type VII alpha subunit	RB276						
Scn8a	Sodium channel voltage-gated type VIII alpha subunit	RB308						

D: Detected (green); ND: Not Detected (yellow)

ESM_1. List of ion channels examined in the P14 rat brain

Rat Gene Symbol	Gene Name	Genepaint Set Id	qPCR Analysis					
			Tissue				Primer	
			Brain	Thymus	Lung	Kidney	Forward	Reverse
Scn9a	Sodium channel voltage-gated type IX alpha subunit	RB277						
Scn10a	Sodium channel voltage-gated type X alpha subunit	RB306						
Scn11a	Sodium channel voltage-gated type XI alpha subunit	RB194						
**CatSper and Two-Pore Channels**								
Catsper1	Cation channel sperm associated 1		ND	ND	ND	ND	cctcaagctaatcgctctgg	ggaggagcacaaagtccaac
Catsper2	Cation channel sperm associated 2	RB293	D	D	D	D	tagaggagcgcaagaggttg	ctggctccaaagacacaggt
Catsper3	Cation channel sperm associated 3		ND	ND	ND	ND	actggatgtggtcattctgg	aggccacagtgtagacggtc
Catsper4	Cation channel sperm associated 4		ND	ND	D	D	gggacgtcatggagaagaag	ggtaatggcgttagtcacca
Tpcn1	Two-pore segment channel 1	RB75	D	D	D	D	acaccatgtttgagctgctg	cccactgaagaactccatgc
Tpcn2	Two-pore segment channel 2	RB76	D	D	D	D	gtggttggtgtcctctgtca	ccaggagctgcttatgacct
**Transient Receptor Potential Channels**								
Trpa1	Transient receptor potential cation channel subfamily A member 1	RB146	D	ND	D	D	cacctttgggcagcttattg	gcttctggacctcagcaatg
Trpc1	Transient receptor potential cation channel subfamily C member 1	RB4	D	D	D	D	gttccaccccacactggtag	gcagtccttctgctctttgg
Trpc2	Transient receptor potential cation channel subfamily C member 2	RB5	D	D	D	D	ggtcacaggctttctgtggt	acaggatgaccacgtccagg
Trpc3	Transient receptor potential cation channel subfamily C member 3		D	ND	D	D	acgaaggtgaactgaaagaa	ccacatttgtgccagagtca
Trpc4	Transient receptor potential cation channel subfamily C member 4	RB147	D	ND	D	D	aatacagtcagccaacgcag	ggaggagcacactctcttgc
Trpc5	Transient receptor potential cation channel subfamily C member 5	RB139	D	D	D	D	tgagaccagagctattgatg	ctcccacaaactccgtgaat
Trpc6	Transient receptor potential cation channel subfamily C member 6	RB140	D	D	D	D	cagcagccaccttatggcta	gccaaccttcttcccttctc
Trpc7	Transient receptor potential cation channel subfamily C member 7	RB18	D	ND	ND	ND	tgaggaattctgaaaacctg	cttgttcaccctcaggtggt
Trpm1	Transient receptor potential cation channel subfamily M member 1	RB19	D	D	D	ND	gaatgcagaagcaccaatgg	aaacgtcttctctatccatg
Trpm2	Transient receptor potential cation channel subfamily M member 2	RB141	D	D	D	D	gatacgtggatgacccaagg	cggtctacaacctgccattc
Trpm3	Transient receptor potential cation channel subfamily M member 3	RB21	D	D	D	D	gagtcgcctctcccgaaatg	ccattccttggtcatcaggt
Trpm4	Transient receptor potential cation channel subfamily M member 4	RB25	D	D	D	D	agttaccgagtggaacagtg	atccgtgcgatcagacagcc
Trpm5	Transient receptor potential cation channel subfamily M member 5	RB22	D	D	D	D	acctggtggtgtccctggtg	actggtcaggatccaggcac

D: Detected (green); ND: Not Detected (yellow)

ESM_1. List of ion channels examined in the P14 rat brain

Rat Gene Symbol	Gene Name	Genepaint Set Id	qPCR Analysis					
			Tissue				Primer	
			Brain	Thymus	Lung	Kidney	Forward	Reverse
Trpm6	Transient receptor potential cation channel subfamily M member 6		ND	ND	D	D	cagcgtcagcaggagttttc	gggtcagaggggaagtctct
Trpm7	Transient receptor potential cation channel subfamily M member 7	RB6	D	D	D	D	gggtgctgctggatataatg	gtccggagagtcttctgtcg
Trpm8	Transient receptor potential cation channel subfamily M member 8	RB37	D	ND	ND	D	ttaccagagactccaaggcc	gttggaaactccttggtgtg
Trpv1	Transient receptor potential cation channel subfamily V member 1	RB142	D	ND	ND	ND	ctgagcttctccctgaggtc	catgtctatctcgagtgctt
Trpv2	Transient receptor potential cation channel subfamily V member 2	RB36	D	D	D	D	ctttcaggctggagacttcc	acactgaagagtcggtcacg
Trpv3	Transient receptor potential cation channel subfamily V member 3		ND	ND	ND	D	ccagaatggctgagaagcag	cagtccacttcacctcgttg
Trpv4	Transient receptor potential cation channel subfamily V member 4	RB26	ND	D	ND	D	agtcctcagtagtgcctggg	ccggttgaagactttgagga
Trpv5	Transient receptor potential cation channel subfamily V member 5		ND	ND	ND	D	agtggcccaggagagggacg	gcttagggatgtagtcctgg
Trpv6	Transient receptor potential cation channel subfamily V member 6	RB27	D	ND	D	D	actcagccttccaccccatg	tgatcagagcctggacattg
Mcoln1	Mucolipin 1	RB2	D	D	D	D	gctgtggaccagtacctgac	agagccagagctgagccatt
Mcoln2	Mucolipin 2	RB32	D	D	D	D	gtccgaacagtggtgactga	ccctcagacactcttccttc
Mcoln3	Mucolipin 3	RB33	D	D	ND	D	agcttctcttagaagatcag	ttcggtccatgtagcctttc
Pkd2	Polycystic kidney disease 2 related	RB3	D	D	D	D	ggcctgggccgagaggctgg	tggagctcatcatgccatag
Pkd2l1	Polycystic kidney disease 2-like 1		ND	ND	ND	ND	agagtccctgtgtccggctg	agactctgacggggtgtgaa
Pkd2l2	Polycystic kidney disease 2-like 2	RB34	D	D	D	D	cagctttccagtccctagcc	accctccatctcggtagacg
Pkdrej	Polycystic kidney disease (polycystin) and REJ homolog (sperm receptor for egg jelly homolog, sea urchin)	RB35	D	D	D	D	ttttgggtacctggtgttcg	tgttctgaaaggctgacacg

Anion channels

Vdac1	Voltage-dependent anion-selective channel protein 1	RB133	D	D	D	D	aagtcccgagtgacccagag	gctgctattccaaagcgagt
Vdac2	Voltage-dependent anion-selective channel protein 2	RB134	D	D	D	D	agatgacctttgacagtgcc	cgagtgcagttggtacctga
Vdac3	Voltage-dependent anion-selective channel protein 3	RB135	D	D	D	D	gacggcgttggttcgagaag	taggttgcctgatgctttcc

Amiloride Receptors

Accn1	Amiloride-sensitive cation channel 1, neuronal	RB325	D	D	D	D	ggagccacgatgagaacatg	ctgtcctaggtcttgggcct
Accn2	Amiloride-sensitive cation channel 2, neuronal	RB326	D	D	D	D	tgggttcgctggccgtcctg	atctgtgtgtccgggatctc
Accn3	Amiloride-sensitive cation channel 3		D	D	D	D	tgctgcctaccttcgtgcac	agttgaaggtgtagcattgcc

D: Detected (green); ND: Not Detected (yellow)

ESM_1. List of ion channels examined in the P14 rat brain

Rat Gene Symbol	Gene Name	Genepaint Set Id	qPCR Analysis					
			Tissue				Primer	
			Brain	Thymus	Lung	Kidney	Forward	Reverse
Accn4	Amiloride-sensitive cation channel 4, pituitary	RB327	D	ND	ND	ND	tgcaggaactgaaggaacag	cttctgtcccaggaatagga
Accn5	Amiloride-sensitive cation channel 5, intestinal		ND	ND	ND	ND	ccggctgtgacattctgtaa	cggaggactttggagactat
Scnn1a	Amiloride-sensitive sodium channel alpha-subunit	RB349	D	D	D	D	gcagctcccgcgacctcctg	tctggttgcacagttggaag
Scnn1b	Amiloride-sensitive sodium channel beta-subunit		ND	D	D	D	atccagacctacctgagctg	acagcctccatcagcttgtc
Scnn1g	Amiloride-sensitive sodium channel gamma-subunit	RB350	D	D	D	D	gcacgttcatgagtcgaaga	acatcccatcgaagaagcag
**Nicotinic Acetylcholine Receptors**								
Chrna1	Cholinergic receptor nicotinic alpha 1 (muscle)	RB328						
Chrna2	Cholinergic receptor nicotinic alpha 2 (neuronal)	RB201						
Chrna3	Cholinergic receptor nicotinic alpha 3 (neuronal)	RB162						
Chrna4	Cholinergic receptor nicotinic alpha 4 (neuronal)	RB163						
Chrna5	Cholinergic receptor nicotinic alpha 5 (neuronal)	RB164						
Chrna6	Cholinergic receptor nicotinic alpha 6 (neuronal)	RB165						
Chrna7	Cholinergic receptor nicotinic alpha 7 (neuronal)	RB166						
Chrna9	Cholinergic receptor nicotinic alpha 9 (neuronal)	RB167						
Chrna10	Cholinergic receptor nicotinic alpha 10 (neuronal)	RB168						
Chrnb1	Cholinergic receptor nicotinic beta 1 (muscle)	RB169						
Chrnb2	Cholinergic receptor nicotinic beta 2 (neuronal)	RB206						
Chrnb3	Cholinergic receptor nicotinic beta 3 (neuronal)	RB170						
Chrnb4	Cholinergic receptor nicotinic beta 4 (neuronal)	RB171						
Chrnd	Cholinergic receptor nicotinic delta (muscle)		ND	D	ND	ND	cacgcatgttctgtctgagg	agctcttcctcccatgacct
Chrne	Cholinergic receptor nicotinic epsilon (muscle)	RB172						
Chrng	Cholinergic receptor nicotinic gamma (muscle)	RB173						
**Dopamine Receptors**								
Drd1a	Dopamine receptor D1	RB329	D	D	D	D	atgccatagagacggtgagc	aaggagccaccacatcagtc

D: Detected (green); ND: Not Detected (yellow)

ESM_1. List of ion channels examined in the P14 rat brain

Rat Gene Symbol	Gene Name	Genepaint Set Id	qPCR Analysis					
			Tissue				Primer	
			Brain	Thymus	Lung	Kidney	Forward	Reverse
Drd2	Dopamine receptor D2	RB330						
Drd3	Dopamine receptor D3	RB331						
Drd4	Dopamine receptor D4	RB332						
Drd5	Dopamine receptor D5	RB333						

GABA-A Receptors

Gabbr1	Gamma-aminobutyric acid (GABA) B receptor, 1	RB289						
Gabbr2	Gamma-aminobutyric acid (GABA) B receptor, 2	RB290						
Gabra1	Gamma-aminobutyric acid (GABA) A receptor alpha 1	RB291						
Gabra2	Gamma-aminobutyric acid (GABA) A receptor alpha 2	RB292						
Gabra3	Gamma-aminobutyric acid (GABA) A receptor alpha 3	RB294						
Gabra4	Gamma-aminobutyric acid (GABA) A receptor alpha 4	RB394						
Gabra5	Gamma-aminobutyric acid (GABA) A receptor alpha 5	RB295						
Gabra6	Gamma-aminobutyric acid (GABA) A receptor alpha 6	RB395						
Gabrb1	Gamma-aminobutyric acid (GABA) A receptor beta 1	RB229						
Gabrb2	Gamma-aminobutyric acid (GABA) A receptor beta 2	RB249						
Gabrb3	Gamma-aminobutyric acid (GABA) A receptor beta 3	RB250						
Gabrd	Gamma-aminobutyric acid (GABA) A receptor delta	RB251						
Gabre	Gamma-aminobutyric acid (GABA) A receptor epsilon	RB252						
Gabrg1	Gamma-aminobutyric acid (GABA) A receptor gamma 1	RB230						
Gabrg2	Gamma-aminobutyric acid (GABA) A receptor gamma 2	RB396						
Gabrg3	Gamma-aminobutyric acid (GABA) A receptor gamma 3	RB254						
Gabrp	Gamma-aminobutyric acid (GABA) A receptor pi		ND	ND	D	D	gtatcccagcaggagacagg	tggaagggacgtaggtttcc
Gabrq	Gamma-aminobutyric acid (GABA) A receptor theta	RB255						
Gabrr1	Gamma-aminobutyric acid (GABA) A receptor rho 1	RB256						

D: Detected (green); ND: Not Detected (yellow)

ESM_1. List of ion channels examined in the P14 rat brain

Rat Gene Symbol	Gene Name	Genepaint Set Id	qPCR Analysis					
			Tissue				Primer	
			Brain	Thymus	Lung	Kidney	Forward	Reverse
Gabrr2	Gamma-aminobutyric acid (GABA) A receptor rho 2	RB397	D	D	D	D	ctggctggctacccaagaag	gtgtgatctggaatagtgtg

Glycine Receptors

Glra1	Glycine receptor alpha-1	RB257						
Glra2	Glycine receptor alpha-2	RB253						
Glra3	Glycine receptor alpha-3	RB309						
Glra4	Glycine receptor alpha-4	RB259						
Glrb	Glycine receptor beta	RB310						

Glutamate Receptors

Gria1	Glutamate receptor ionotropic AMPA1	RB273						
Gria2	Glutamate receptor ionotropic AMPA2	RB274						
Gria3	Glutamate receptor ionotropic AMPA3	RB399						
Gria4	Glutamate receptor ionotropic AMPA4	RB275						
Grid1	Glutamate receptor ionotropic delta 1	RB320						
Grid2	Glutamate receptor ionotropic delta 2	RB321						
Grik1	Glutamate receptor ionotropic kainate 1	RB322						
Grik2	Glutamate receptor ionotropic kainate 2	RB323						
Grik3	Glutamate receptor ionotropic kainate 3	RB324						
Grik4	Glutamate receptor ionotropic kainate 4	RB148						
Grik5	Glutamate receptor ionotropic kainate 5	RB149						
Grin1	Glutamate receptor ionotropic NMDA1	RB269						
Grin2a	Glutamate receptor ionotropic NMDA2A	RB150						
Grin2b	Glutamate receptor ionotropic NMDA2B	RB232						
Grin2c	Glutamate receptor ionotropic NMDA2C	RB270						
Grin2d	Glutamate receptor ionotropic NMDA2D	RB233						

D: Detected (green); ND: Not Detected (yellow)

ESM_1. List of ion channels examined in the P14 rat brain

Rat Gene Symbol	Gene Name	Genepaint Set Id	qPCR Analysis					
			Tissue				Primer	
			Brain	Thymus	Lung	Kidney	Forward	Reverse
Grin3a	Glutamate receptor ionotropic NMDA3A	RB271						
Grin3b	Glutamate receptor ionotropic NMDA3B	RB272	D	D	D	D	gcttccagctgtggtgaact	ggccctgaaatgaggtgttg
Grina	Glutamate receptor ionotropic N-methyl D-aspartate-associated protein 1	RB400						
Grm1	Glutamate receptor metabotropic 1	RB236						
Grm2	Glutamate receptor metabotropic 2	RB143						
Grm3	Glutamate receptor metabotropic 3	RB144						
Grm4	Glutamate receptor metabotropic 4	RB234						
Grm5	Glutamate receptor metabotropic 5	RB403						
Grm6	Glutamate receptor metabotropic 6	RB145						
Grm7	Glutamate receptor metabotropic 7	RB235						
Grm8	Glutamate receptor metabotropic 8	RB404						

5-HT3 (Serotonin) Receptors

Htr1a	5-hydroxytryptamine (serotonin) receptor 1A	RB174						
Htr1b	5-hydroxytryptamine (serotonin) receptor 1B	RB377						
Htr1d	5-hydroxytryptamine (serotonin) receptor 1D	RB405						
Htr1f	5-hydroxytryptamine (serotonin) receptor 1F	RB378						
Htr2a	5-hydroxytryptamine (serotonin) receptor 2A	RB379						
Htr2b	5-hydroxytryptamine (serotonin) receptor 2B		ND	ND	D	D	actgcctccatcatgcatct	attcctttaatagggactgg
Htr2c	5-hydroxytryptamine (serotonin) receptor 2C	RB380						
Htr3a	5-hydroxytryptamine (serotonin) receptor 3A, ionotropic	RB370						
Htr3b	5-hydroxytryptamine (serotonin) receptor 3B, ionotropic	RB371						
Htr4	5-hydroxytryptamine (serotonin) receptor 4, G protein-coupled	RB151						
Htr5a	5-hydroxytryptamine (serotonin) receptor 5A, G protein-coupled	RB372						
Htr6	5-hydroxytryptamine (serotonin) receptor 6, G protein-coupled	RB373						

D: Detected (green); ND: Not Detected (yellow)

ESM_1. List of ion channels examined in the P14 rat brain

Rat Gene Symbol	Gene Name	Genepaint Set Id	qPCR Analysis					
			Tissue				Primer	
			Brain	Thymus	Lung	Kidney	Forward	Reverse
Htr7	5-hydroxytryptamine (serotonin) receptor 7, adenylate cyclase-coupled	RB374						
**Inositol Receptors**								
Itpr1	Inositol 1,4,5-trisphosphate receptor type 1	RB375	D	D	D	D	accgggatgccccatcccgg	atcagggtcggcctccctcc
Itpr2	Inositol 1,4,5-trisphosphate receptor type 2	RB258	D	D	D	D	agaagaaagacagcttcatg	cttgtgctgtcttggcgtag
Itpr3	Inositol 1,4,5-trisphosphate receptor type 3	RB191	D	D	D	D	cagctaaggaagatgctgct	agtcctgatccacgccagag
**Purinergic Receptors P2X**								
P2rx1	P2X purinoceptor 1	RB312	D	D	D	D	tgtgcagagaacccagaagg	tcacatgtcttcacagtgcc
P2rx2	P2X purinoceptor 2	RB301	D	D	D	D	gctccttcctgtgtgactgg	gtggatgtggagtcctgttg
P2rx3	P2X purinoceptor 3	RB302	D	ND	D	D	ggaccattgggatcatcaac	cacgtccatgactctgttgg
P2rx4	P2X purinoceptor 4	RB303	D	D	D	D	agacgccgactgcactcctg	ccaacgtcgttctccaccgg
P2rx5	P2X purinoceptor 5	RB260						
P2rx6	P2X purinoceptor 6	RB261						
P2rx7	P2X purinoceptor 7	RB304	D	D	D	D	ggcaccatcaagtggatctt	ccctccgtgacattctctgt
**Ryanodine Receptors**								
Ryr1	Ryanodine receptor 1	RB192						
Ryr2	Ryanodine receptor 2	RB356						
Ryr3	Ryanodine receptor 3	RB193						
**Chloride Channels**								
Clca1	Calcium-activated chloride channel 1							
Clca2	Calcium-activated chloride channel 2		ND	D	ND	ND	acctctcccagcaggctctg	tcgttgccaatggtactgtc
Clca3	Calcium-activated chloride channel 3		ND	ND	D	ND	agctgaagatgggatctttg	gagaggcctgagtcaccatg
Clca4	Calcium-activated chloride channel 4		ND	D	D	D	gaagttgtccttgggcagag	tagccacggtctctgcattc
Clca5	Calcium-activated chloride channel 5	RB237	D	D	D	D	aggggcaaaccacaagctat	attagccccgacgagttcac
Clcn1	Chloride channel, voltage-sensitive 1	RB238						

D: Detected (green); ND: Not Detected (yellow)

ESM_1. List of ion channels examined in the P14 rat brain

Rat Gene Symbol	Gene Name	Genepaint Set Id	qPCR Analysis					
			Tissue				Primer	
			Brain	Thymus	Lung	Kidney	Forward	Reverse
Clcn2	Chloride channel, voltage-sensitive 2	RB244						
Clcn3	Chloride channel, voltage-sensitive 3	RB245						
Clcn4	Chloride channel, voltage-sensitive 4	RB246						
Clcn5	Chloride channel, voltage-sensitive 5	RB247						
Clcn6	Chloride channel, voltage-sensitive 6	RB248						
Clcn7	Chloride channel, voltage-sensitive 7	RB391						
Clcnka	Chloride channel, voltage-sensitive ClC-Ka	RB392						
Clcnkb	Chloride channel, voltage-sensitive ClC-Kb	RB264	D	D	D	D	agtgagtcagcctgggagtc	gatgtgacagaacgcattgg
Clic1	Chloride intracellular channel 1		D	D	D	D	atcccgggccgtgagacccg	ttggcgccatcactcccagc
Clic2	Chloride intracellular channel 2		D	D	D	D	caagcttagcgctcaacacc	cgttggcagaaaggacagtt
Clic3	Chloride intracellular channel 3	RB265	D	ND	D	D	ccatcctactctatgatggg	ggtgccaggccaggaaagtc
Clic4	Chloride intracellular channel 4	RB266	D	D	D	D	atggcgctgtcgatgcccct	ttcctatgctctcaccatca
Clic5	Chloride intracellular channel 5	RB227	D	D	D	D	tgcaaggtgatggacaaatg	aggccttcattcacgctaag
Clic6	Chloride intracellular channel 6	RB228	D	ND	D	ND	atggacgtagggagaacggc	gagacgctgtgagaatgggc

Other Potassıum Channels

Rat Gene Symbol	Gene Name	Genepaint Set Id	Brain	Thymus	Lung	Kidney	Forward	Reverse
Kcnip1	Kv channel interacting protein 1	RB212						
Kcnip2	Kv channel interacting protein 2	RB213	D	D	D	D	agcgccctattctggccacc	agcagcttgaggaaacgctg
Kcnip3	Kv channel interacting protein 3	RB214	D	ND	ND	ND	gttgctgcttaatcaagtgg	agctggtccaagccctctgg
Kcnip4	Kv channel interacting protein 4	RB215	D	D	D	D	cacggaccacaatggagctg	tgtagccatctttgtttatg
Kctd1	Potassium channel tetramerisation domain containing 1	RB216	D	D	D	D	actgctcctccggcattacc	gttgttcagtggcgatgcgg
Kctd2	Potassium channel tetramerisation domain containing 2	RB210	D	D	D	D	atacgggacaacgagaacag	tgattgctctcgtccttcag
Kctd3	Potassium channel tetramerisation domain containing 3	RB211	D	D	D	D	atcgtgcagctgaatgttgg	ttctgcctcgtgtctgagaa
Kctd4	Potassium channel tetramerisation domain containing 4	RB187	D	D	D	D	ccggaggagttctctgtgtc	tgaagagcatcgctgtgaac
Kctd5	Potassium channel tetramerisation domain containing 5	RB188	D	D	D	D	gccctggctcagcgccccag	ggtggggtctctgtcgatta

D: Detected (green); ND: Not Detected (yellow)

ESM_1. List of ion channels examined in the P14 rat brain

Rat Gene Symbol	Gene Name	Genepaint Set Id	qPCR Analysis					
			Tissue				Primer	
			Brain	Thymus	Lung	Kidney	Forward	Reverse
Kctd6	Potassium channel tetramerisation domain containing 6	RB189	D	D	D	D	gacgcctggagatgccactg	tatccatctgctccagcact
Kctd7	Potassium channel tetramerisation domain containing 7	RB190	D	D	D	D	agactcggagggtcggtact	catgttctccagctgctcca
Kctd8	Potassium channel tetramerisation domain containing 8		D	D	D	D	accgagacgacaagatctgg	tcacagctggatgtggagag
Kctd9	Potassium channel tetramerisation domain containing 9	RB205	D	D	D	D	atttaagccgctgcaacctc	ttacagagcctcagggatgc
Kctd10	Potassium channel tetramerisation domain containing 10		D	D	D	D	cagccgtgaagctgctctac	aagcgcagggagagcttatc
Kctd11	Potassium channel tetramerisation domain containing 11		D	D	D	D	gcttaaggatgggtcctctg	cctagtgctcaaccctccag
Kctd12	Potassium channel tetramerisation domain containing 12		D	D	D	D	atggctctggcggacagcac	acggacaccacggtacagcg
Kctd13	Potassium channel tetramerization domain containing 13	RB208	D	D	D	D	acagtctgaagcccctgacc	agttgagaattgtaccaaag
Kctd14	Potassium channel tetramerisation domain containing 14	RB209	D	D	D	D	atatacggtggcctctcagc	ccaccacaggctctaacgat
Kctd15	Potassium channel tetramerisation domain containing 15	RB240	D	D	D	D	aaggcctggggcttccaatg	accgggtgagtgacagccgg
Kctd16	Potassium channel tetramerisation domain containing 16	RB241	D	D	D	ND	catggtggcctgtaactcct	agtctggatgttgccctgac
Kctd17	Potassium channel tetramerisation domain containing 17	RB242	D	D	D	D	acggcctgagttcggagtcc	ccgcctccacctgcacctgg
Kctd18	Potassium channel tetramerisation domain containing 18	RB243	D	D	D	D	accacttggccaatgagatg	atggcatctgtcccatcagt
Kctd19	Potassium channel tetramerisation domain containing 19		ND	ND	D	D	gagctttgtgcctttctgga	gtatatagtgacctctgctg
Kctd20	Potassium channel tetramerisation domain containing 20	RB239	D	D	D	D	aggtagctctcaggccccag	aatccagaactgtgcggaac
Kctd21	Potassium channel tetramerisation domain containing 21		ND	ND	ND	ND	agcctttggtgggcaaggag	ggcatcttcccactgaacat

Auxiliary Subunits

Abcc4	ATP-binding cassette, Multidrug resistance-associated protein 4	RB362	D	D	D	D	ccaggacttgcattcagagg	aacctgcccagcatccaagg
Abcc8	ATP-binding cassette, sub-family C (CFTR/MRP), member 8	RB136						
Abcc9	ATP-binding cassette, sub-family C (CFTR/MRP), member 9	RB137						
Anxa7	Annexin A7	RB363	D	D	D	D	cctccctggctccatattgc	aggtgggtagcctgtcgggg
Bsnd	Bartter syndrome, infantile, with sensorineural deafness (Barttin)	RB364						
Cftr	Cystic fibrosis transmembrane conductance regulator (ABCC7)		ND	ND	D	D	cagatcaggttggcctcaag	tcgctcgaatcacttggtatg
Cul5	Cullin-5	RB365	D	D	D	D	aactagaggtgactttattg	aacataggactctcgcactc

D: Detected (green); ND: Not Detected (yellow)

ESM_1. List of ion channels examined in the P14 rat brain

Rat Gene Symbol	Gene Name	Genepaint Set Id	qPCR Analysis					
			Tissue				Primer	
			Brain	Thymus	Lung	Kidney	Forward	Reverse
Cybb	Cytochrome B-245 heavy chain	RB366	D	D	D	D	gtggagtggtgtgtgaatgc	cgagtcacagccacatacag
Fxyd1	FXYD domain-containing ion transport regulator 1	RB367	D	D	D	D	ccacaccctgcggatcggcg	ttggttgaatttgcaccggc
Fxyd2	FXYD domain-containing ion transport regulator 2	RB393	D	D	D	D	gacggagaatcccttcgagt	tgctgaggagaatgaggagt
Fxyd3	FXYD domain-containing ion transport regulator 3	RB369	D	D	D	D	cctgttggtccttctagcag	agagaatccctgcacagatg
Gck	Glucokinase (hexokinase 4)	RB407						
Grinl1a	GRINL1A complex locus 1	RB376						
Mtap1a	Microtubule-associated protein 1A	RB381						
Mink1	Misshapen-like kinase 1	RB398						
Ms4a2	Membrane-spanning 4-domains, subfamily A, member 2	RB401	D	D	D	D	ctgagcaacaactccgctta	tcatcggggaccttactacg
Nola1	GAR1 ribonucleoprotein homolog (yeast)	RB402	D	D	D	D	gaggtggtggaagaggtggt	atgtccccttcctcggaatc
Nox1	NADPH oxidase homolog 1	RB217	D	D	D	D	tactctgacctctgctccag	ggaagacatcctcactgact
Nudt9	ADP-ribose pyrophosphatase, mitochondrial precursor	RB231	D	D	D	D	cgtaactgtccggtcctcag	cttcgctcgacttttgaacc
Psmd1	Proteasome (prosome, macropain) 26S subunit, non-ATPase, 1	RB218						
Sh3kbp1	SH3-domain binding protein 1	RB219	D	D	D	D	atggcagacgaggagtgttt	agtgtttccggtctttcagg
Shkbp1	SH3KBP1 binding protein 1	RB220	D	D	D	D	gacttcttctcgggcctctt	gattcccaagactggtggag
Slc12a5	Solute carrier family 12, (potassium-chloride transporter) member 5	RB221						
Stx1b	Syntaxin-1B1	RB382	D	ND	ND	ND	cgtaactgtccggtcctcag	cttcgctcgacttttgaacc
Tnfaip1	Tumor necrosis factor, alpha-induced protein 1, endothelial		ND	ND	ND	ND	aggccagaggccgcacggag	agattaccacacggcaggag
Tomm40	Translocase of outer mitochondrial membrane 40 homolog (yeast)	RB262	D	D	D	D	gtggcctgggcactgggtca	gtatgggtcacctggaaacg
Tpte	Transmembrane phosphoinositide 3-phosphatase and tensin homolog	RB263	D	ND	ND	ND	tgttcttctccgagtgtatg	taaatgacagccacgagcac

D: Detected (green); ND: Not Detected (yellow)
