# Supplementary material for: A Digital Atlas of Ion Channel Expression Patterns in the Two-Week-Old Rat Brain
Source: Neuroinformatics. 2014 Oct 7;13(1):111–25. doi: 10.1007/s12021-014-9247-0 (PMC4303740; doi:10.1007/s12021-014-9247-0)
Supplement: Supplementary file 2 — (PDF 4460 kb) [file 12021_2014_9247_MOESM2_ESM.pdf]

A Digital Atlas of Ion Channel Expression Patterns in the Two-Week-Old Rat Brain

Volodymyr Shcherbatyy[1], James Carson[2], Murat Yaylaoglu[1], Katharina Jäckle[1], Frauke Grabbe[1], Maren Brockmeyer [1], Halenur Yavuz [1], and Gregor Eichele[1*]

[1] *Department of Genes and Behavior, Max Plank Institute for Biophysical Chemistry, Göttingen, Germany*
[2] *Life Sciences Computing, Texas Advanced Computing Center, Austin, TX, USA*

[*] Correspondence to: Gregor Eichele, Department of Genes and Behavior, Max Planck Institute for Biophysical Chemistry, Am Fassberg 11, 37077 Göttingen, Germany
E-mail address: gregor.eichele@mpibpc.mpg.de

Online Resource 2

ESM_2. Subdivision mesh atlas of the P14 rat brain.

Twenty seven meshes with a color legend indicating the 13 major brain structures present in the subdivision mesh atlas.

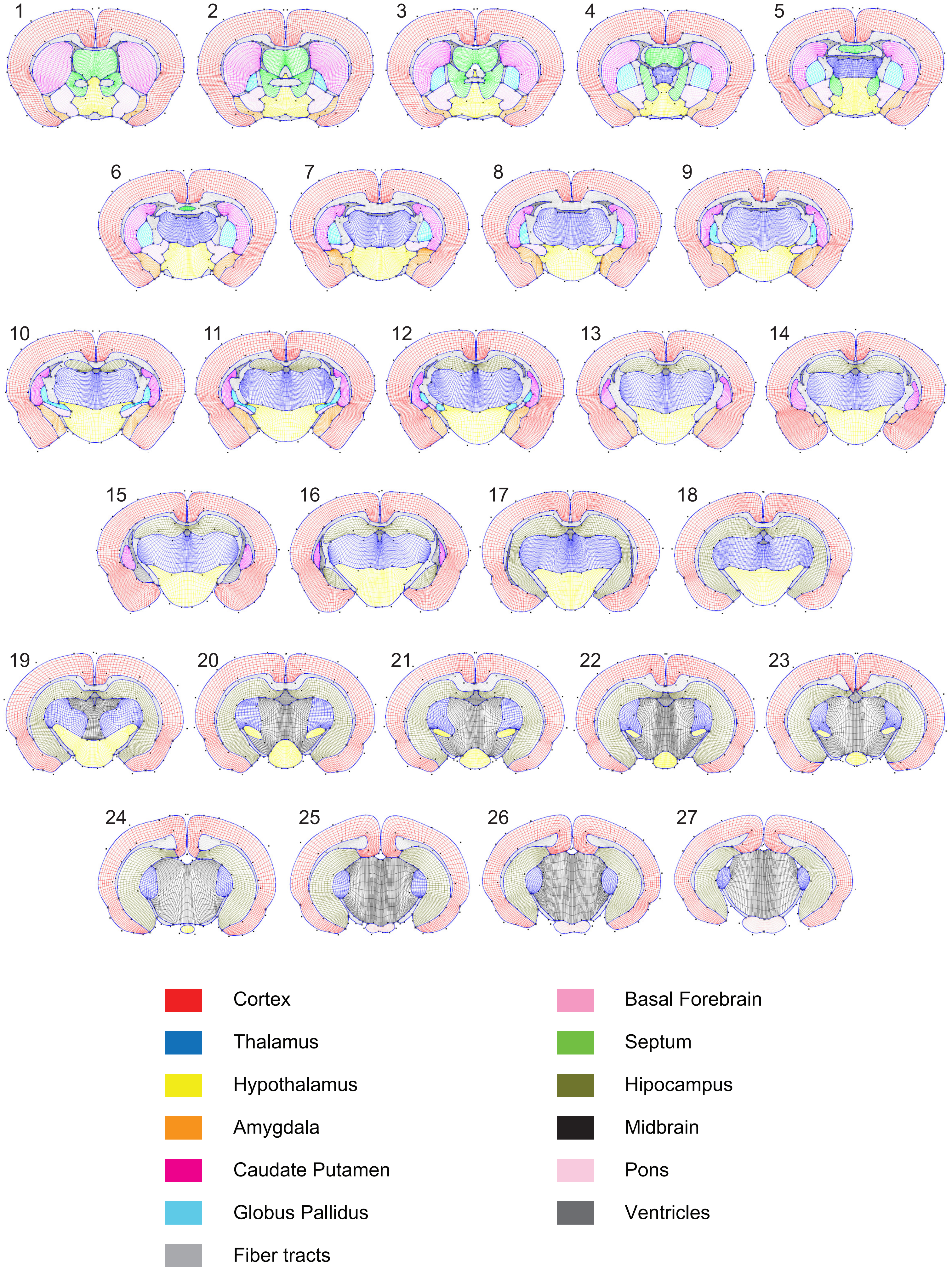
1
2
3
4
5
6
7
8
9
10
11
12
13
14
15
16
17
18
19
20
21
22
23
24
25
26
27
Cortex
Thalamus
Hypothalamus
Amygdala
Caudate Putamen
Globus Pallidus
Fiber tracts
Basal Forebrain
Septum
Hipocampus
Midbrain
Pons
Ventricles
